# Wood Nutrient-Water-Density Linkages Are Influenced by Both Species and Environment

**DOI:** 10.3389/fpls.2022.778403

**Published:** 2022-04-04

**Authors:** Demetrius Lira-Martins, Carlos Alberto Quesada, Stanislav Strekopytov, Emma Humphreys-Williams, Bruno Herault, Jon Lloyd

**Affiliations:** ^1^Department of Life Sciences, Imperial College London, London, United Kingdom; ^2^Department of Plant Biology, Institute of Biology, University of Campinas (UNICAMP), Campinas, Brazil; ^3^Coordination of Environmental Dynamics, National Institute for Amazonian Research (INPA), Manaus, Brazil; ^4^Imaging and Analysis Centre, Natural History Museum, London, United Kingdom; ^5^National Measurement Laboratory, LGC, Teddington, United Kingdom; ^6^UR Forests and Societies, Centre de Coopération Internationale en Recherche Agronomique Pour le Développement (Cirad), Montpellier, France; ^7^Université de Montpellier, Montpellier, France; ^8^Institut National Polytechnique Félix Houphouët-Boigny, Yamoussoukro, Ivory Coast; ^9^Centre for Tropical, Environmental and Sustainability Sciences, College of Science and Engineering, James Cook University, Smithfield, QLD, Australia; ^10^Faculdade de Filosofia, Ciencias e Letras de Ribeirão Preto, Universidade de São Paulo, Ribeirão Preto, Brazil

**Keywords:** ecophysiology, cations, potassium, tropical forests, water storage, wood traits, wood density, nutrients

## Abstract

Tropical trees store a large amount of nutrients in their woody tissues, thus triggering the question of what the functional association of these elements with other wood traits is. Given the osmotic activity of mineral elements such as potassium, sodium, and calcium, these elements should be strong candidates in mediating the water storing capacity in tropical trees. We investigated the role of wood nutrients in facilitating wood water storage in trees by using branch samples from 48 tropical tree species in South America and examined their associations with wood density (ρ). Wood density varied from 316 kg/m^3^ in Peru plots, where the soil nutrient status is relatively higher, to 908 kg/m^3^ in Brazil plots, where the nutrient availability is lower. Phosphorus content in wood varied significantly between plots with lowest values found in French Guiana (1.2 mol/m^3^) and plots with highest values found in Peru (43.6 mol/m^3^). Conversely, potassium in woody tissues showed a significant cross-species variation with *Minquartia guianensis* in Brazil showing the lowest values (8.8 mol/m^3^) and with *Neea divaricata* in Peru having the highest values (114 mol/m^3^). We found that lower wood density trees store more water in their woody tissues with cations, especially potassium, having a positive association with water storage. Specific relationships between wood cation concentrations and stem water storage potential nevertheless depend on both species’ identity and growing location. Tropical trees with increased water storage capacity show lower wood density and have an increased reliance on cations to regulate this reservoir. Our study highlights that cations play a more important role in tropical tree water relations than has previously been thought, with potassium being particularly important.

## Introduction

Plants employ a plethora of ecological strategies to facilitate the acquisition of light, water, CO_2_, and mineral nutrients necessary for their growth, reproduction, and dispersal. These strategies should be reflected in physiological and morphological features indicating differences between species in terms of their adaptive design. For example, wood density (ρ) is considered to reflect a range of hydraulic functionalities (Chave et al., 2009), with high-density wood being linked to increased hydraulic safety (Hacke et al., 2001; Jacobsen et al., 2005; Pratt et al., 2007, 2021; Jansen et al., 2011) and an associated capacity for maintaining operational functionality at lower water potentials (Ackerly, 2004; Bucci et al., 2004; Santiago et al., 2004; Jacobsen et al., 2007; Pratt et al., 2021). At the other end of the spectrum, several studies have noted a negative association between wood water storage capacity and ρ (Borchert and Pockman, 2005; Scholz et al., 2007; Richards et al., 2014; Oliva Carrasco et al., 2015; Fu and Meinzer, 2019; Chaturvedi et al., 2021).

Plants can rely substantially on the stem water reservoir to buffer daily transpiration (Holbrook and Sinclair, 1992; Goldstein et al., 1998; Blackman et al., 2019; Preisler et al., 2021). Moreover, as transpiration creates negative hydraulic pressures, surrounding tissues should, if hydrated, be able to provide water to the vascular tissues to reduce the risk of embolism. The structural mechanisms responsible for enhancing the water storage capacity in trees are underpinned by the anatomical configuration of the woody tissues with both capillary storage compartments (i.e., open vessels, tracheids, fibers, intercellular spaces, and cracks) and elastic storage compartments (i.e., living parenchyma cells) likely to be relevant (Borchert and Pockman, 2005; Scholz et al., 2008; Jupa et al., 2016; Kawai et al., 2021). Not only the anatomic features define plant water storing capacity but also the chemical compounds with osmotic activity play an essential role in plant osmotic adjustment (Turner and Jones, 1980; Turner, 2018; Signori-Müller et al., 2021). The accumulation of osmotically active elements in the tissues decreases the water potential, thus enabling the maintenance of high cellular turgor potential and water retention (Blum, 2017; Traversari et al., 2020; da Silva et al., 2021). Soluble carbohydrates act as osmolytes and control the cell pressure by decreasing the osmotic potential from cells and increasing the water retention, thus avoiding tissue water deficit (Hartmann and Trumbore, 2016; Ozturk et al., 2021; Tomasella et al., 2021); but, these are rather metabolically expensive use for these metabolites compared with ionic solutes (Leigh and Wyn Jones, 1984; Sevanto et al., 2014). Given the important roles played by mineral nutrients such as potassium and sodium as osmotically active solutes in both leaves and roots (Benlloch-González et al., 2008; Shabala and Shabala, 2011; Battie-Laclau et al., 2014; Tavakol et al., 2018; da Silva et al., 2021), inorganic cations, in general, are strong candidates for evaluation as potential facilitators of water storage in woody tissues. For instance, K and Ca have been indicated as strong drivers of water balance in the xylem of poplar trees (Traversari et al., 2020). Furthermore, K is known to have a positive effect on wood formation by driving the osmotic potential of the cambium (Fromm, 2010).

In this study, to test the hypothesis that trees with increased internal water storage should have higher concentrations of osmotically active ions within their tissues, we examined associations between wood water content, wood nutrients, and wood density using branch material collected from moist tropical forests in Brazil, French Guiana, and Peru. We expect any observed trait variation to be mediated not only by changes in soil nutrient status and climate but also by species turnover in response to these environmental properties (Fyllas et al., 2009; Patiño et al., 2009; Schrodt et al., 2015). Therefore, we used a mixed-effect (MEM) model approach (Lira-Martins et al., 2019) to specifically probe whether relationships between stem water content, wood density, and stem nutrient concentrations also vary according to both species’ identity and growing conditions.

## Materials and Methods

### Study Sites

**FIGURE 1:**
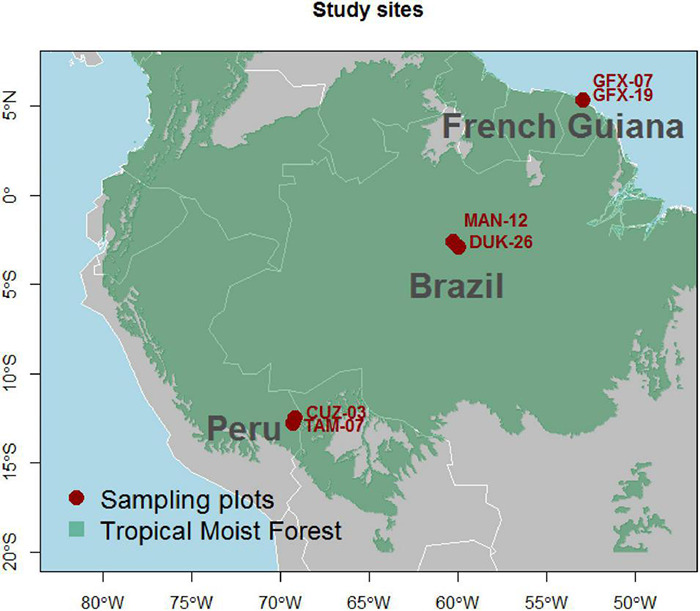
Location of the six sampling plots.

We sampled a total of six plots of 1 ha with two “paired” plots in each of Brazil, French Guiana, and Peru (Figure 1) chosen based on contrasting soil chemical properties. Climatic conditions of the plot pairs were similar within each country (Supplementary Table 1).

### Sampling

Within each plot, professional tree climbers trimmed one branch sample from the upper canopy of the selected trees, all of which were of diameter at breast height greater than 10 cm, and we sampled in total 2–12 branches per species with each branch representing a single tree. We attempted to consistently sample fully sun-exposed branches close to the canopy apex. For each plot, the most abundant species were identified before sampling, and a minimum of two trees per species were sampled. We also included in the sampling design species that were locally less abundant but were found across the studied plots. This assured a minimal overlap of species across plots, thus allowing us to disentangle the influence of species from the local environmental effects on the assessed traits. A total of 48 species were sampled in this study (Supplementary Table 2) with 6 species overlapping across plots. All of the species in the study have ring diffuse porosity.

### Wood Trait Determinations

After collection, branches were kept inside plastic bags and brought back to the field laboratory for processing. This was usually on the same day, but when not possible, samples were wrapped in humid paper towels, sealed in plastic bags, and stored in the fridge for processing within 24 h. For the determination of wood density, water content, and nutrient concentration, we trimmed a piece of *ca.* 20 cm length and *ca.* 2 cm diameter from the sampled branch. This 20 cm piece was divided in two parts, with one sample used for nutrient determinations, and the other retained for wood density and water content measurements. Since we were mostly interested in xylem properties of wood, bark and pith greater than 2 mm diameter were removed from all samples prior to analysis. We did not use the same piece of wood for all measurements as rehydration of the piece can potentially rinse off nutrients, especially mobile ones such as potassium. Nevertheless, we were aware of wood density variation along the branch (Longuetaud et al., 2017), and therefore, we used these consecutive pieces assuming their lack of independence to represent the branch traits.

#### Wood Density and Water Content

Branch segments allocated for wood density and water content were soaked in water for 24 h, blotted dry, and weighed on a precision balance. Their hydrated volume (υ_*s*_) was then measured by the water displacement method (Archimedes’ principle). Samples were then dried in a fan oven at 105°C until constant weight, usually 2 days, with wood density calculated as:


(1) $$\rho =\frac{m_{d}}{\upsilon_{s}}$$

where *m*_*d*_ is the dry mass of the sample (kg) and υ_*s*_ is the volume of the hydrated sample (m^3^).

Although previous studies examining relationships between wood water content and wood density have expressed the former on a dry weight basis (Lima and Rodal, 2010), a more parsimonious approach that also serves to avoid spurious correlations (Lloyd et al., 2013) is to consider wood density, wood nutrient concentrations, and wood water content variations all on a per unit tissue volume basis. In this study, water content is thus expressed as the hydrated water content per unit tissue volume and is estimated as follows:


(2) $$\Theta =\frac{\upsilon_{w}}{\upsilon_{s}}=\frac{m_{s}-m_{d}}{m_{d}}.\rho$$

where Θ is the hydrated volumetric water content (m^3^/m^3^), υ_*w*_ is the volume of water (cm^3^) of an assumed density of 1,000 kg/m^3^, and *m*_*s*_ is the mass of the saturated sample (kg).

#### Nutrient Determinations

Wood samples were first dried for 48 h at 65°C, after which they were ground in a planetary ball mill (Retsch PM 400). For the determination of Ca, K, Mg, Na, and P, 200 mg of the finely ground material was digested in a microwave system (CEM MARS X) using XP1500 Plus fluoropolymer vessels with 5 ml of concentrated nitric acid and 0.5 ml of hydrogen peroxide (both of trace element analysis grade). Certified reference material of Willow wood (WEPAL-IPE-220) was run every three batches with 11 samples in each batch. The extracts were then diluted to 50 ml in ultrapure water and subsequently analyzed using ICP-OES (Thermo iCap 6500 Duo). For all elements analyzed, reference material values were within the stated uncertainty limits.

Nitrogen analyses were undertaken using a CHN elemental analyser Vario EL cube (Elementar Analysensysteme GmbH). For each measurement of N per tree, we used 5 mg of wood material. For each tree, we ran three replicates of the same sample to ensure consistency of N determination. Certified Birch Leaf (Catalog code B2166) material and Acetanilide standards (Catalog code B2000), both from Elemental Microanalysis Ltd., United Kingdom, were used to check the accuracy of the N analysis. The ICP-OES and N analyses were undertaken at the Imaging and Analysis Centre at the Natural History Museum in London, United Kingdom, with the digestions having been carried out at Imperial College London—Silwood Park Campus, United Kingdom. Element concentration in wood is given as follows:


(3) $$\Theta_{m}=\frac{m_{\Theta }}{m_{d}}$$

where Θ_*m*_ represents the nutrient amount per unit tissue dry mass with values given by the nutrient amount, *m*_Θ_ (mol), in an amount of tissue of dry weight, *m*_*d*_ (g). The nutrient content per tissue volume Θ_*v*_ (mol/m^3^) was then calculated as follows:


(4) $$\Theta_{v}=\frac{m_{\Theta }}{m_{s}}=\Theta_{m}.\rho$$

### Statistical Analyses

We employed a mixed effect model (MEM) accounting for each tree (*t*) being located within a specific sampling plot (*p*) and of a known species identity (*s*). To examine the sources of variability of the assessed traits, a null multilevel model was first fitted at the log_10_ transformed data. This model deals with this hierarchy structure by using crossed random factors (Snijders and Bosker, 2012) as follows:


(5) $$Wt_{\left(p,s\right)}=\gamma_{00}+U_{0p}+V_{0s}+R_{tp}$$

We used the same notation as in Snijders and Bosker (2012) and Lira-Martins et al. (2019), where *Wt*_*(p,s)*_ represents one of the evaluated wood traits of each individual of known species identity located within a given plot, γ_00_ is the overall intercept, *U*_*0p*_ is a random variable that quantifies the variance associated with sampling location (i.e., the plot effect), *V*_0*s*_ is the random variation associated with species identity (i.e., the species effect), and *R*_*tp*_ represents the residual variance. These random effects were computed by the best linear unbiased predictor (BLUP) that provides estimates from the distances between the terms and the grand means (Zuur et al., 2009). The *R*_*tp*_, *U*_0*p*_, and *V*_0*s*_ terms are assumed to be drawn from normally distributed populations, and the variance of the residuals (*R*_*tp*_) is assumed to be independent of species or sampling location. Individual proportions of each term were calculated as the variance of each term divided by the sum of all terms, i.e., *R*_*tp*_ + *U*_0*p*_ + *V_0s_*.

To examine the associations of nutrients with wood density (ρ) and water content (Φ) in wood, Equation 5 was extended to accommodate an independent variable as follows:


(6) $$Wt_{\left(p,s\right)}=\gamma_{00}+\gamma_{10}X_{tp}+U_{0p}+V_{0s}+R_{tp}$$

where γ_10_ is the overall regression coefficient quantifying the relationship between the evaluated trait and the independent variable *X*_*tp*_ for any given tree. In all cases, traits had been log_10_ transformed to help meet linear model assumptions of a normal distribution of residuals.

All MEM model fits were carried out using the lme4 package (Bates et al., 2015) available within the R (3.4.3) statistical platform (R Core Team., 2018). Associated probability values were extracted using the lmerTest package (Kuznetsova et al., 2017), and to assess the significance of the random intercept terms, a restricted likelihood ratio test was implemented using the function exactRLRT from package RLRsim (Scheipl et al., 2008). This test simulates values from a finite distribution of simultaneous tests, thereby providing an exact likelihood ratio test. We also tested for the significance of random slopes by using the same function. The r.squaredGLMM function from the MuMin package (Barton, 2018) was also used to quantify the marginal and conditional *R*^2^. These can be considered to represent the variation explained by the fixed effects and by the whole model (including the random effects), respectively (Nakagawa and Schielzeth, 2013).

## Results

### Intraspecific, Interspecific, and Between Plot Wood Trait Variation

**FIGURE 2:**
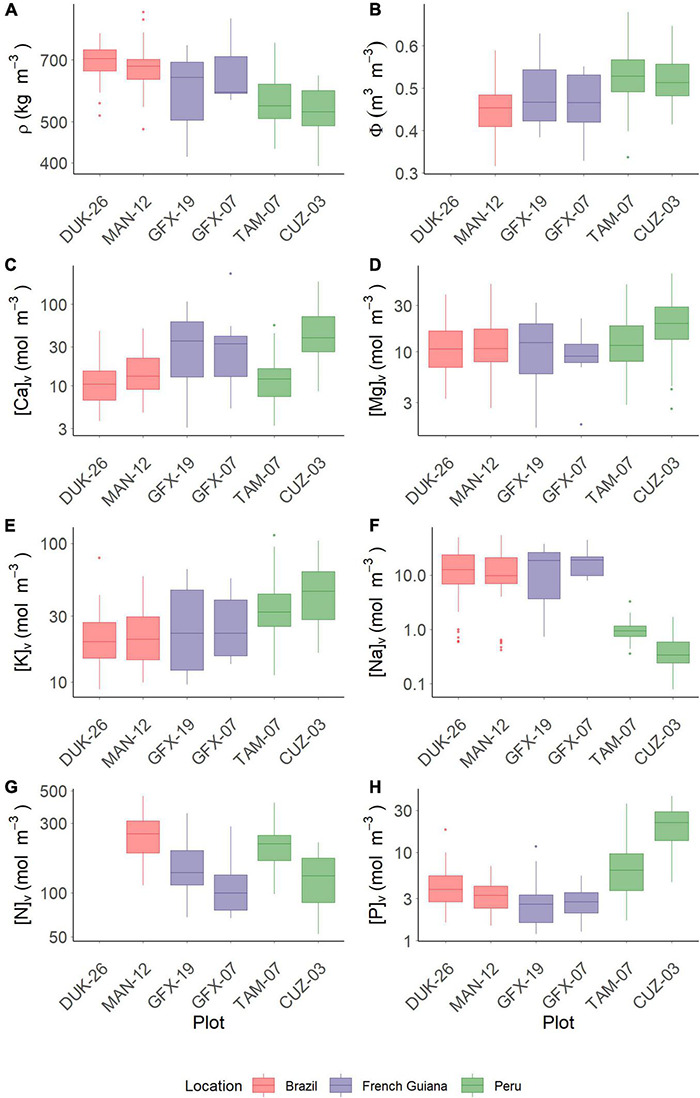
Variation of traits in each studied plot. **(A)** Wood density (ρ). **(B)** Water content per unit tissue volume (Φ). **(C)** Calcium. **(D)** Magnesium. **(E)** Potassium. **(F)** Sodium. **(G)** Nitrogen. **(H)** Phosphorus. Data on Φ and N are not available for DUK-26.

The plot shown in Figure 2 denotes the data ranges observed for branch wood density (ρ), branch water content (Φ), and associated concentrations of Ca, Mg, K, Na, N, and P when expressed on a volume basis. The lowest ρ value was observed for the two Peruvian sites (Figure 2A), where the lowest value reached 316 kg/m^3^, whereas the highest value was found in Brazil (908 kg/m^3^) (Supplementary Table 3). Conversely, branch volumetric water contents showed the opposite pattern. The MAN-12 and the French Guiana sites showed similar patterns (Figure 2B) with no significant differences detected between them (Supplementary Table 3). For [Ca]_*v*_, the Brazilian sites and TAM-07 (Peru) had the lowest values, with the French Guiana sites being intermediate and CUZ-03 (Peru) being the highest (Figure 2C). Contrasting that pattern was magnesium content in wood, with an overall between-plot variability being much less than for Ca content in wood, but with [Mg]_*v*_ for the Peruvian CUZ-03 plot being, on average, higher than for the plots in Brazil and French Guiana (Figure 2D). Potassium content in wood was higher in the Peruvian sites, where *Neea divaricata* showed the highest value (114 mol/m^3^), and with little difference between the other four sites (Figure 2E), where the Brazilian *Minquartia guianensis* showed the lowest value (8.8 mol/m^3^). Sodium concentrations in branch tissues were *ca.* 10-fold lower in Peru as compared with both Brazil and French Guiana (Figure 2F). For nitrogen, a Peru vs. Brazil/French Guiana contrast was less apparent, with the highest values found for the Brazilian MAN-12 and the Peruvian TAM-07 (Figure 2G). Phosphorus content in branch tissues showed little variation amongst sites in Brazil and French Guiana, with the latter having the lowest value (1.2 mol/m^3^). The two Peruvian sites had the highest P content in wood (43.6 mol/m^3^), and they were significantly higher than the other sites (Figure 2H). Supplementary Table 3 further summarizes the data underlying Figure 2 with the results of a similar analysis using the more conventional dry-mass-based nutrient metrics. Essentially the results are the same irrespective of whether nutrients are expressed on a dry-mass or volumetric basis. Nevertheless, site-to-site differences in the former sometimes assume a higher level of significance. For example, when expressed on a dry-mass basis, there is a difference in potassium content in wood between the two Peruvian sites (*P* = 0.008), but this is not the case when potassium content is expressed on a volumetric basis (*P* = 0.062).

**FIGURE 3:**
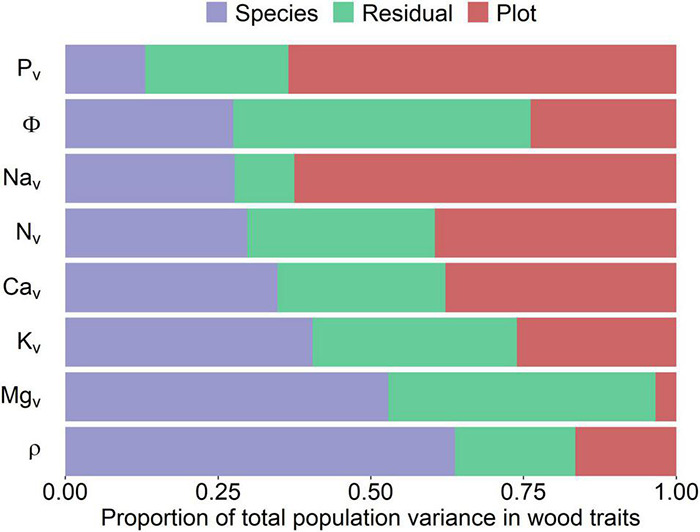
Partitioning of the total variance for each woody property (*y*-axis) into species, environmental (plot), and error (residual) components. Traits have been ranked according to the relative contribution of the species-associated variance component. All values were log_10_ transformed prior to analysis with nutrients on a volumetric basis being used. Subscript “v” indicates that concentrations are on a volume basis. ρ, wood density; Φ, water content per unit tissue volume.

Figure 3 shows a partitioning of variance as quantified through Equation 5. This shows the proportion of the total trait variance explained by species and environment varying substantially according to the trait. For example, species identity explained most of the variation in ρ (0.59) and [Mg]_*v*_ (0.55), with the effect of plot location explaining only 0.16 and 0.05 of the total variances, respectively. In contrast, sampling location explained most of the variance in [P]_*v*_ (0.65), [Na]_*v*_ (0.60), and [Ca]_*v*_ (0.43). In terms of the residual term, which should include intra-species variation and measurement sampling error, this component was largest for Φ (0.60) and [Mg]_*v*_ (0.39). For consistency, we also employed the same approach for data on a mass basis (Supplementary Figure 1), with results indicating that nutrients on a mass basis followed basically the same patterns as in a volumetric basis with only subtle differences between the two metrics.

### Nutrient Associations With Wood Density

The magnitude and significance of the within-tree associations between wood nutrient concentrations and ρ as determined by the MEM are shown in Table 1. For all six nutrients examined, likelihood ratio tests indicated significant random intercept terms associated with both species identity (*V*_0*s*_) and plot location (*U*_0*p*_) (*P* = 0.012, results not shown). Nevertheless, in no case were random slope effects detected (*P* = 0.109, results not shown). Only [K]_*v*_ showed any sort of statistically meaningful association with ρ with a slope of −0.55 ± 0.27 (*P* = 0.046). Although the marginal *R*^2^, metric quantifying only the variation explained by the fixed effect component of the model (*R*_*M*_^2^), was only 0.03, the conditional *R*^2^ of the model was much higher (*R*_*C*_^2^ = 0.65). This indicates that the systematic variations between species and across sites form a critically important part of the ρ: [K]_*v*_ association. Indeed, an analysis of the random terms indicates that with σ_*s*_^2^ = 0.026, species identity accounted for 0.43 (i.e., 0.026/(0.026 + 0.012 + 0.022) of the total variance not accounted for by the fixed term. A similar calculation for plot location yields an estimate of 0.20.

**TABLE 1:** Mixed effect modeling of the relationships between nutrients expressed on a tissue volume basis (Θ_*v*_) and wood density (ρ).

Θ_*v*_ (mol/m^3^) vs. Wood density ρ (kg/m^3^)						
Nutrient	[Ca]_*v*_	[K]_*v*_	[Mg]_*v*_	[N]_*v*_	[Na]_*v*_	[P]_*v*_
**Fixed effects**						
Intercept (γ_00_)	0.45	2.96	1.71	1.00	1.80	1.82
Slope (γ_10_)	0.31	−**0.55**	–0.22	0.45	–0.45	–0.41
*P*	0.456	0.046	0.537	0.121	0.370	0.218
$R_{\text{M}}^{2}$	0.00	0.03	0.00	0.02	0.00	0.01
$R_{\text{C}}^{2}$	0.74	0.65	0.56	0.67	0.91	0.75
**Random effects**						
Species (σ_*s*_^2^)	0.062	0.026	0.049	0.014	0.166	0.021
Plot (σ_*p*_^2^)	0.070	0.012	0.003	0.017	0.382	0.093
Residual (σ_*r*_^2^)	0.047	0.022	0.041	0.016	0.057	0.037

*Subscript “v” indicates that concentrations are on a volume basis.*

*σ^2^ represents the variance of each term.*

*All parameters were estimated using restricted maximum likelihood (REML).*

*Significant relationships (P < 0.05) are in bold.*

*Random effects present the variance of the random intercepts (Equation 6).*

*$R_{M}^{2}$, marginal R^2^ that represents the variation explained by the fixed effects; $R_{\text{C}}^{2}$, conditional R^2^ that represents the variation of the whole model (including the random effects).*

For comparative purposes, modeled associations with nutrients expressed on a mass rather than a volume basis are shown in Supplementary Table 4. In all cases, the fixed effect slope estimates were exactly 1.0 unit more negative for Θ_*m*_ than Θ_*v*_, and due to the same standard errors, in most cases, the mass-based metrics present a higher level of statistical significance. The mathematics underlying the consistent differences between the mass vs. volume-based slopes (despite the same standard errors) is presented in Supplementary Information 2. Also, for any given nutrient random variance, partitioning results and fitted intercepts were identical for the Θ_*m*_ vs. Θ_*v*_ models.

**FIGURE 4:**
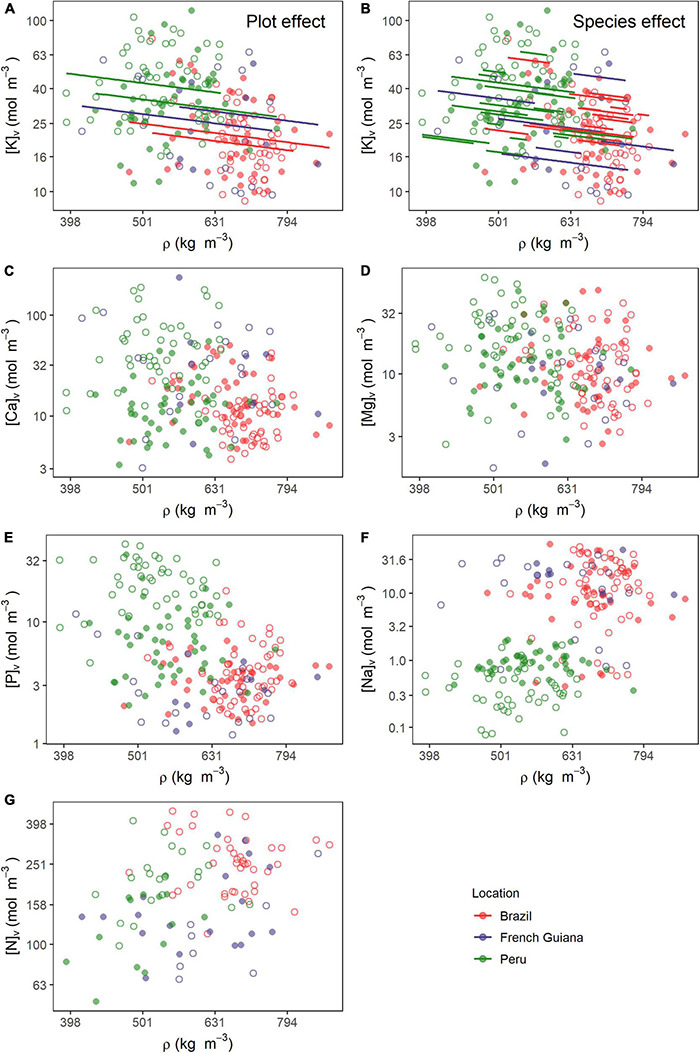
Relationships between wood density (ρ) and nutrients on a volume basis (Θ_*v*_). Lines in **(A)** and **(B)** are predicted estimates of mixed models for the species and plot effects (Equation 6) for the relationship between ρ and potassium. Relationships of ρ with **(C)** calcium, **(D)** magnesium, **(E)** phosphorus, **(F)** sodium, and **(G)** nitrogen. All variables were log10 transformed prior to analysis. Open and closed circles represent the plots within each location.

Figure 4 shows in graphical form the data underlying the analyses of Table 1 with fitted relationships indicated where the fixed slope estimate was significant. This shows, for example, the two plots in Peru tend to have a higher [K]_*v*_ at any given ρ than do those of Brazil (Figure 4A). Figure 4B shows the same data, but in this case with the species effects displayed. This shows that superimposed on the general negative relationship, there should be *ca.* fourfold differences between species average [K]_*v*_ at any given ρ. But after accounting for the generally lower ρ in Peru, there is no clear systematic difference in the spectrum of [K]_*v*_ vs. ρ associations for species typically found in Peru vs. those found in Brazil and/or French Guiana. This can be observed when comparing the within-country range of variation in the [K]_*v*_ vs. ρ relationship (Figure 4B), which are similar across countries.

Although according to the MEM analysis, relationships between wood density ρ and [Ca]_*v*_ (Figure 4C), [Mg]_*v*_ (Figure 4D), [P]_*v*_ (Figure 4E), [Na]_*v*_ (Figure 4F), and [N]_*v*_ (Figure 4G) were not significant at *P* = 0.05 or better, when the data for all plots and species are simply pooled and analyzed using OLS, all five of these elements show apparent relationships with ρ at *P <* 0.001. Correlation coefficients ranged from *R*^2^ = 0.06 for [Mg]_*v*_ to *R*^2^ = 0.26 for [Na]_*v*_ (Supplementary Table 5) with the latter relationship being especially interesting. This is because a simple examination of Figure 4F suggests that there is less, if any, relationship between [Na]_*v*_ vs. ρ within plots and thus with the biased OLS relationship simply being due to trees in plots with higher overall ρ also tending to have a higher [Na]_*v*_.

### Wood Density/Water Associations

**FIGURE 5:**
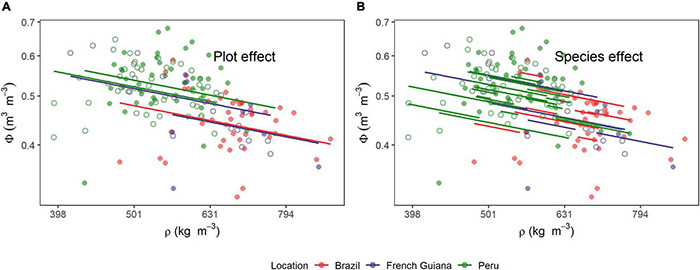
Relationships between wood density (ρ) and stem volumetric water content (Φ). Lines are predicted estimates of mixed models for the **(A)** species and **(B)** plot effects (Equation 6). Open and closed circles represent the plots within each location.

The MEM model results for the relationship between water content in wood (Φ) with ρ are shown in Table 2, with underlying data and model predictions shown in Figure 5. Together these show a strong negative within-plot association between ρ and Φ (*P* < 0.001) but also with a large proportion of the variation in water content not accountable by variations in wood density, as indicated by the larger $R_{C}^{2}$ (0.57) than $R_{M}^{2}$ (0.12). Systematic differences between species and plots were also modest as can be seen by the large variance in the residuals (σ^2^ = 0.0019) as compared with the species (σ^2^ = 0.0012) and plot variance (σ^2^ = 0.005) terms. Overall, the slope of −0.38 ± 0.07 suggests a less than 1:1 replacement of dry matter by water as wood density declines. For example, given the log transformation of the variables, the model predicts that, on average, a doubling in wood density is associated with a [1 − 2^(–0.38)^] = 23% decrease in wood volumetric water content. For consistency, we also evaluated the association of ρ with water content on a mass basis. This relationship showed a slope of −1.39 ± 0.07 (*P* < 0.001, Supplementary Figure 2).

**TABLE 2:** Mixed effect modeling of relationships between wood density (ρ) and water content per unit tissue volume (Φ).

	ρ *vs.* Φ
**Fixed effects**	
Intercept	0.73
Slope	−**0.38**
*P*	<0.001
$R_{\text{M}}^{2}$	0.12
$R_{\text{C}}^{2}$	0.57
**Random effects**	
Species (σ_*s*_^2^)	0.0012
Plot (σ_*p*_^2^)	0.0005
Residual (σ_*r*_^2^)	0.0019

*All parameters were estimated using restricted maximum likelihood (REML).*

*σ^2^ represents the variance of each term.*

*$R_{M}^{2}$, marginal R^2^ that represents the variation explained by the fixed effects; $R_{\text{C}}^{2}$, conditional R^2^, which represents the variation of the whole model (including the random effects).*

*Significant relationships (P < 0.05) are mentioned in bold.*

*Random effects present the variance of the random intercepts and slope (Equation 6).*

### Wood Nutrient/Water Association

**FIGURE 6:**
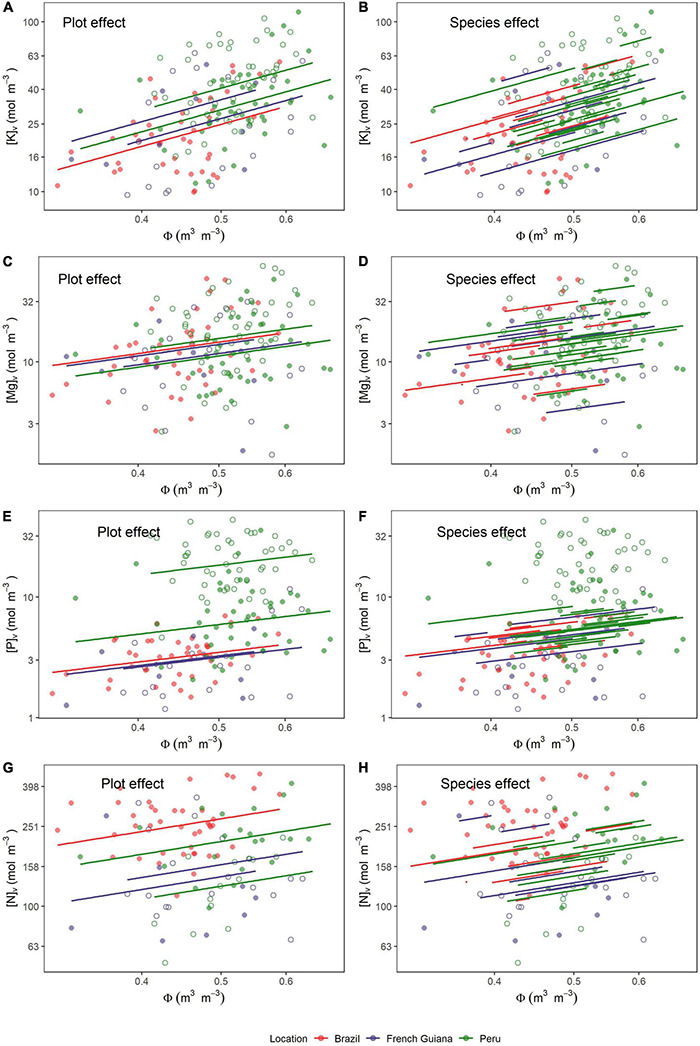
Relationships between water content (Φ) and nutrients on a volume basis (Θ_*v*_). Lines are predicted estimates of mixed models for the species effect and plot effect (Equation 6) for **(A,B)** potassium, **(C,D)** magnesium, **(E,F)** phosphorus, and **(G,H)** nitrogen. Open and closed circles represent the plots within each location.

The magnitude and statistical significance of the within-tree associations between wood nutrient concentrations and wood water content as determined are shown in Table 3 with the underlying data presented in Figure 6. As for the nutrient wood vs. density associations, likelihood ratio tests indicated that although for all six nutrients there was significant random intercept terms associated with both species identity and plot location (*P* < 0.004, data not shown), in no case were random slope effects detected (*P* > 0.098, data not shown).

**TABLE 3:** Mixed effect modeling of the relationships between nutrients expressed on a tissue volume basis (Θ_*v*_) and water content (Φ).

Θ_*v*_ (mol/m^3^) *vs.* Water content Φ (m^3^/m^3^)						
Nutrient	[Ca]_*v*_	[K]_*v*_	[Mg]_*v*_	[N]_*v*_	[Na]_*v*_	[P]_*v*_
**Fixed effects**						
Intercept (γ_00_)	1.59	1.89	1.42	2.44	0.70	0.97
Slope (γ_10_)	0.71	**1.34**	**0.98**	**0.66**	0.67	**0.84**
*P*	0.102	<0.001	0.008	0.012	0.175	0.014
$R_{\text{M}}^{2}$	0.01	0.13	0.04	0.04	0.00	0.02
$R_{\text{C}}^{2}$	0.71	0.68	0.61	0.74	0.91	0.76
**Random effects**						
Species (σ_*s*_^2^)	0.061	0.023	0.052	0.017	0.133	0.015
Plot (σ_*p*_^2^)	0.059	0.009	0.006	0.021	0.502	0.107
Residual (σ_*r*_^2^)	0.050	0.020	0.038	0.014	0.063	0.041

*Subscript “v” indicates that concentrations are on a volume basis.*

*All parameters were estimated using restricted maximum likelihood (REML).*

*σ^2^ represents the variance of each term.*

*$R_{M}^{2}$, marginal R^2^ that represents the variation explained by the fixed effects; $R_{\text{C}}^{2}$, conditional R^2^ that represents the variation of the whole model (including the random effects).*

*Significant relationships (P < 0.05) are in bold.*

*Random effects present the variance of the random intercepts (Equation 6).*

Overall, potassium was the nutrient showing the strongest association with Φ with a fixed effect slope of 1.3 ± 0.26 (*P* < 0.001). As can be seen through a comparison of Figures 6A,B, the species random term accounted for the highest fraction of the random variance component, this being over twice that associated with plot identity (Table 3). Magnesium also showed a positive association with Φ (slope = 0.98 ± 0.37; *P* = 0.008) with much of the variance not accounted for by the fixed effect component attributable to species identity (Figures 6C,D and Table 3). Also, [P]_*v*_ showed a positive association with Φ (slope = 0.84 ± 0.34; *P* = 0.014). However, in contrast to K and Mg, it was sampling location (Figure 6E) rather than species identity (Figure 6F) which was the dominant random variance term (Table 3). In addition, [N]_*v*_ showed a positive association with Φ (slope = 0.66 ± 0.26; *P* = 0.012) with similar variance apportionments to both plot and species identity (Figures 6G,H and Table 3). Finally, [Ca]_*v*_ and [Na]_*v*_ also showed positive relationships with Φ (Supplementary Figures 3A,B), but with neither of these being significant at *P* < 0.05.

Again, for comparison, we analyzed the same relationships, but with the nutrients expressed on a mass rather than a volume basis (Supplementary Table 6). In all cases, relationships showed slightly steeper slopes when expressed on a mass basis and, in most cases, with marginally higher *R*_*M*_^2^ and *R*_*C*_^2^ as well.

### Associations Between Random Effects

**FIGURE 7:**
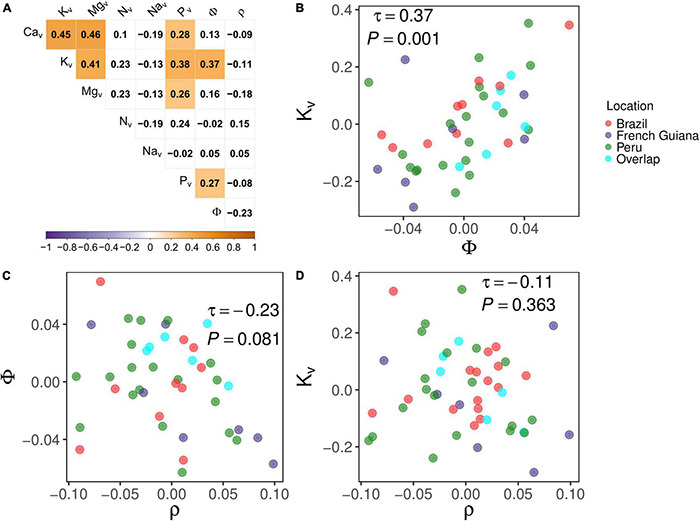
**(A)** Correlation matrix between species effects (Equation 5) as given by Kendall’s τ with *P*-values adjusted as per Benjamini and Hochberg (1995). Subscript “v” indicates that concentrations are on a volume basis. Φ, volumetric wood water content; ρ, wood density. **(B)** Relationship between species effects of Φ and K_*v*_ (Equation 5). **(C)** Relationship between species effects of Φ and ρ. **(D)** Relationship between species effects of ρ and K_*v*_. Cyan circles in panels **(A–C)** depict species that occur in more than one location.

For each trait examined, the individual species effect term of Equation 5 (i.e., *V*_0*s*_) provides an estimate of the differences between each species and the grand mean of that trait (see section “Wood Trait Determinations”). Thus, using the non-parametric Kendall’s τ as a measure of associations, the strength of these “species effects” relationships is shown in Figure 7A. This suggests a strong species-dependent positive association of [K]_*v*_ with tissue water content (τ = 0.37, *P* = 0.001) as shown in Figure 7B. This occurred despite the lack of any sort of strong association between ρ and Φ (τ = −0.23, *P* = 0.081, Figure 7C) or between ρ and [K]_*v*_ (τ = −0.11, *P* = 0.363, Figure 7D). For the interested reader, individual species effects are detailed in Supplementary Table 2. This shows, for example, that, even after accounting for differences in growing conditions, *N. divaricata* and *Matisia bicolor* (in this study found growing in Peru) and *Helicostylis tomentosa* (in this study found growing in Brazil) are typically characterized by higher-than-average values of both [K]_*v*_ and Φ. On the contrary, *Bocoa prouacensis*, *Licania heteromorpha*, and *Lecythis persistens*—all sampled in French Guiana in this study—typically have lower-than-average values for both [K]_*v*_ and Φ. These strong species influences are further illustrated for [K]_*v*_ in Supplementary Figure 4 and for Φ in Supplementary Figure 5 using a “box and whisker” approach. In these figures, observed values for each species are ranked from the lowest to the highest median value with a separate panel for each studied plot.

Given that we only had six plots, the statistical power to test the relationships between individual plot random effect terms was limited. Thus, there were no correlations at *P* = 0.05 or better. Similarly, due to the small sample size, we could evidence systematic associations at *P* = 0.05 or between any of the plot-level random effects and any of the climate/soil variables as listed in Supplementary Table 1.

## Discussion

For the analysis in this study, we used branch samples to examine the interrelationships between wood nutrient concentrations, wood water content and wood density with all entities expressed on a volumetric basis. We have also undertaken all analysis with nutrients and/or water on a dry mass basis in Supplementary Information 2. The structural parameters in woody tissues tend to vary along the main axis of the tree (Dória et al., 2019; Olson et al., 2021) and also across their radial dimension (Björklund et al., 2017; Longuetaud et al., 2017; Rathgeber, 2017). Furthermore, given the biomechanical differences between branches and stems, structural differences can be expected with potential impact on the slopes and intercepts of the evaluated relationships. Nevertheless, given the strong associations between branch and stem (Swenson and Enquist, 2008; Baraloto et al., 2010; Sarmiento et al., 2011; He and Deane, 2016), we expect the relationships shown in this study to be commonly found within woody tissues and, therefore, to also hold for woody stem tissues.

### Contrasting Sources of Variation for Different Nutrients

When analyzed in the simplest way *via* the null model of Equation 5, the different wood traits studied showed contrasting underlying sources of variation. For example, Figure 3 suggests that much of the variation in ρ was attributable to species identity, a result consistent with this trait being associated with many aspects of the life history of trees, namely, growth and mortality rates (Chao et al., 2008; Chave et al., 2009; Poorter et al., 2010). Conversely, variation in Φ was partitioned almost equally between species and the environment but with the residual variance component still representing over 50% of the total (Figure 3). Thus, the amount of water per unit volume in woody tissues seems to vary dramatically within any one species.

The partitioning of the variance for [N]_*v*,_ [Ca]_*v*_, and [K]_*v*_ also showed broadly similar contributions of the species and sampling location. In contrast, the strong contribution of species identity to the observed variations in [Mg]_*v*_ suggests a strong phylogenetic constraint on wood Mg concentrations. This has also been found to be the case for foliar tissues (Broadley et al., 2004, 2008; Fyllas et al., 2009; Asner et al., 2012; White et al., 2015; Asner and Martin, 2016) and with a strong association between foliar and woody [Mg] in tropical tree species (Lira-Martins et al., 2019).

In contrast with the above elements, variations in [P]_*v*_ and [Na]_*v*_ were mainly driven by plot location, i.e., [P]_*v*_ varied more between plots than between species and therefore with different species in the same plot tending to have similar [P]_*v*_. Although high [Na] in plant tissues seems to be phylogenetically conserved (White et al., 2017), in this study variation in [Na]_*v*_ was strongly driven by sampling location. Although trees growing closer to the coast should receive an increased input of Na from the coast (Stallard and Edmond, 1981; Vet et al., 2014), this does not help explain the relatively high [Na]_*v*_ for the central Amazonian DUK-26 and MAN-12 sites. However, we noted that, interestingly, relatively high aerosol sodium concentrations of an apparently fungal origin have been recently reported in this area (China et al., 2018).

### Wood Density and Nutrients

Only [K]_*v*_ showed a significant relationship with ρ at *P* = 0.05 or better (Table 1). This relationship was negative suggesting that lighter woods are characterized by higher concentrations of this nutrient per unit volume within their cell walls and/or living cells. Nevertheless, the random variance component associated with species identity was also large, and as can be seen from Figure 4B, different species varied in their [K]_*v*_: ρ relationship. Even once differences in wood density are accounted for, there exists significant systematic differences between different species in their characteristic potassium concentrations per unit wood volume.

Although none of the other elements showed significant associations with wood density when expressed on a volume basis, when the same nutrients were expressed on a dry mass basis, then significant negative slopes were found for magnesium and phosphorus (Supplementary Table 4) with the fixed effect slope of all elements examined being exactly 1.0 unit more negative than on a mass basis (Supplementary Information 2). Although the functional significance of the negative [K]_*v*_:ρ association remains unclear, it is interesting to note that Quesada et al. (2012) found a strong negative relationship between soil exchangeable potassium concentrations and tropical forest stand level wood densities across the Amazon Basin. This raises the interesting possibility that one factor accounting for the tendency for higher wood density trees to dominate on relatively dystrophic soils is their overall lower potassium requirement per unit volume growth increment. Nevertheless, the fixed effect slope of the [K]_*v*_: ρ relationship of −0.55 ± 0.27 is relatively modest meaning, for example, that a halving of wood density requires, on average (for any given species located in any given plot), an increase in [K]_*v*_ of [1 − 0.5^(–0.55)^] or just 46%.

Interestingly, it should be noted that tropical trees with low [K] in wood also show low levels of this nutrient in foliar tissues (Heineman et al., 2016; Lira-Martins et al., 2019) with this potentially impacting plant stomata aperture. [K] plays an important role in guard cell osmoregulation described for leaves. Potassium content in guard cells increases in parallel with early morning opening so that stomatal opening is associated primarily with K+ uptake (Taiz et al., 2015). Trees that need less [K] to open their stomata would have a benefit over trees that need higher [K], especially on soils lacking high [K].

### Relationships Between Wood Density and Wood Water Contents

Although Φ and ρ were negatively associated (Figure 5), this is hardly surprising as water and dry matter must (along with air) necessarily compete for the limited space within a given stem tissue volume. Nevertheless, when comparing across species, there was no convincing negative correlation between Φ and ρ (Figure 7C), suggesting that a lower wood density does not necessarily imply a higher amount of water when species contrasts constitute the sole source of variation. Perhaps this is because space in a given volume not occupied by mass nor water will be filled by gas, which can itself be considered a costless filler, with the mechanical advantage of not unnecessarily overloading the stems of taller trees (Gartner et al., 2004; Poorter, 2008; Dias et al., 2020). Hence, the volume in the wood of low-density trees can be filled by gas or water, with the substantial osmotic potential associated with high cation concentrations being one likely essential prerequisite for the successful deployment of the latter strategy.

### Water Content and Wood Nutrients

Overall, our MEM analysis showed a significant increase in the nutrient tissue concentration with water content Φ and with [Ca]_*v*_ and [Na]_*v*_ being the only elements that did not vary systematically with Φ. For the Φ:[K]_*v*_ and Φ:[Mg]_*v*_ associations, as is evidenced by the relatively high random species associated variances (Table 3), differences between species identity were also clearly important. This suggests that although similar relationships exist across all species, individual species tend to differ in their characteristic “operating points” (Figures 6B,D). This adds information to the intra-specific variation of Φ detected by the variance partitioning (Figure 3) and suggests that variations in the water content within the species may be partially explained by the variation in cations, especially [K]_*v*_. The positive association between the species random effects of Φ and [K]_*v*_ (Figure 7B) indicates that, other things being equal, species with higher than average [K]_*v*_ typically also have a higher-than-average wood tissue water content. That a somewhat similar pattern observed for P (Figure 7A) further demonstrates that internal water storage differences may underpin a suite of physiological mechanisms taking place within woody tissues. Indeed, all of the cations studied have well-documented roles acting as osmolytes in plant tissues (Hawkesford et al., 2012; Battie-Laclau et al., 2014, 2016; Blum, 2017; da Silva et al., 2021), with inorganic P potentially acting as a counterbalance anion, at least in some circumstances (Jeschke et al., 1997).

The existence of significant random plot effects (Table 3) indicates that, even after accounting for species differences, there was still an effect of growing location on many nutrient vs. Φ associations. This was especially the case for nitrogen (Figure 6G), phosphorus, and, to a lesser extent, potassium (Figure 6A). Although there was no significant correlation with Φ for both [Ca]_*v*_ and [Na]_*v*_, there was still evidence of systematic plot-to-plot variation in these elements (Figure 2) which would be useful to understand. Nevertheless, as we only had six plots within our dataset, we lacked sufficient statistical power to identify the underlying climatic and/or edaphic drivers behind these effects of sampling location.

## Conclusion

Our study brings new insights into the nutritional associations with wood density and water storage in woody tissues of tropical trees by using branch samples. Specifically, the evaluation of the underlying causes for the observed covariation between these traits indicates that some of them are most strongly influenced by species identity, whilst others are primarily determined by environmental conditions. Potassium was the only element showing a significant association with wood density. Furthermore, the ability of tropical woods to store water is clearly linked with wood nutrient contents with K apparently being the most important cation in this respect.

## Data Availability Statement

The data that support the findings of this study are available from the corresponding author, DL-M, upon reasonable request.

## Author Contributions

DL-M and JL designed the study and wrote the manuscript. DL-M, EH-W, and SS developed the laboratory protocols and analyzed the wood samples. DL-M conducted the fieldwork and carried out all the statistical analyses. CQ provided the Peruvian and Brazilian soil data. BH provided the logistical support in French Guiana. All authors edited the manuscript and approved its current version.

## Conflict of Interest

The authors declare that the research was conducted in the absence of any commercial or financial relationships that could be construed as a potential conflict of interest.

## Publisher’s Note

All claims expressed in this article are solely those of the authors and do not necessarily represent those of their affiliated organizations, or those of the publisher, the editors and the reviewers. Any product that may be evaluated in this article, or claim that may be made by its manufacturer, is not guaranteed or endorsed by the publisher.
