# Supplementary material for: Wood Nutrient-Water-Density Linkages Are Influenced by Both Species and Environment
Source: Front Plant Sci. 2022 Apr 4;13:778403. doi: 10.3389/fpls.2022.778403 (PMC9014131; doi:10.3389/fpls.2022.778403)
Supplement: Supplementary file 1 [file Data_Sheet_1.docx]

**Table S1** Environmental and soil characteristics (0- 30 cm depth) of the study sites

| **Country** | | **Brazil** | | **French Guiana** | | | **Peru** | | | |
| --- | --- | --- | --- | --- | --- | --- | --- | --- | --- | --- |
| Plot | DUK-26 | | MAN-12 | | GFX-19† | GFX-07†† | | CUZ-03 | TAM-07 |  |
| Longitude | -59.94 | | -60.21 | | -52.92 | -52.92 | | -68.96 | -69.26 |  |
| Latitude | -2.95 | | -2.61 | | 5.3 | 5.3 | | -12.50 | -12.83 |  |
| Elevation | 110 | | 106 | | NA | NA | | 203 | 218.9 |  |
| WRB Soil Group | Ferrasols | | Ferrasols | | Acrisol | Acrisol | | Cambisols | Cambisols |  |
| Soil pH | 4.27 | | 3.94 | | 4.2 | 4.81 | | 6.07 | 4.22 |  |
| Sand fraction | 12.71 | | 19.9 | | 47.8 | 80 | | 5.26 | 47.01 |  |
| Silt fraction | 40.79 | | 12.39 | | 9 | 5.3 | | 52.27 | 24.2 |  |
| Clay fraction | 46.5 | | 67.71 | | 43.2 | 15.7 | | 42.46 | 28.79 |  |
| Nitrogen (g kg^-1^) | 1.7 | | 1.5 | | N.D. | 1.24 | | 2.4 | 1.4 |  |
| [Ca]_total_ (mmol kg^-1^) | 10.88 | | 0.27 | | N.D. | 0.09 | | 167.41 | 2.75 |  |
| [Mg]_total_ (mmol kg^-1^) | 4.77 | | 3.74 | | N.D. | 0.10 | | 495.99 | 74.74 |  |
| [K]_total_ (mmol kg^-1^) | 3.95 | | 0.95 | | N.D. | 0.20 | | 466.1 | 93.72 |  |
| [Na]_total_ (mmol kg^-1^) | 4.7 | | 0 | | N.D. | N.D. | | 95.74 | 13.91 |  |
| [P]_total_ (mmol kg-1) | 54.97 | | 101.78 | | N.D. | 0.8 | | 727.02 | 178.22 |  |
| [Ca]_ex_ (mmol kg^-1^) | 0.48 | | 1.1 | | N.D. | 0.09 | | 49.5 | 0.4 |  |
| [Mg]_ex_ (mmol kg^-1^) | 0.4 | | 1 | | N.D. | N.D. | | 16.7 | 1 |  |
| [K]_ex_ (mmol kg^-1^) | 0.38 | | 0.9 | | N.D. | N.D. | | 2 | 0.8 |  |
| [Na]_ex_ (mmol kg^-1^) | 0.6 | | 0.6 | | N.D. | N.D. | | 0.2 | 0.3 |  |
| [P]_available_ (mmol kg-1) | N.D. | | 12.92 | | N.D. | N.D. | | 46.13 | 21.65 |  |
| *P*_A_ (mm) | 2193 | | 2325 | | 2755 | 2755 | | 2150 | 2458 |  |
| MAP (°C) | 27.1 | | 27.1 | | 25.9 | 25.9 | | 25.3 | 25.4 |  |
| Sampling date | Feb-16 | | Mar-16 | | Jan-17 | Jan-17 | | Jun-16 | Jul-16 |  |

[Ca]_total_, [Mg]_total_, [K]_total_, [Na]_total_ = total soil calcium, magnesium, potassium, sodium and phosphorus. [Ca]_ex_, [Mg]_ex_, [K]_ex_ and [Na]_ex_ = exchangeable calcium, magnesium, potassium and sodium concentrations respectively; [P]_available_= available phosphorus, *P*_A_ = mean annual precipitation; MAP = Mean annual temperature, N.D. = not determined. Soil data for Brazil Peru is from Quesada *et al.* (2010) with climate data obtained from the interpolated WorldClim dataset at 2.5 minutes spatial resolution (Hijmans, Cameron, Parra, Jones & Jarvis 2005). †Soil data from Hättenschwiler & Jørgensen (2010). ††Soil data from Epron *et al.* (2006)

**Table S2.** Species effects obtained by Eqn. 5 for each studied trait.

| Species | Country | [Ca]_v_ | [K]_v_ | [Mg]_v_ | [N]_v_ | [Na]_v_ | [P]_v_ | *Φ* | *ρ* |
| --- | --- | --- | --- | --- | --- | --- | --- | --- | --- |
| *Conceveiba guianensis* | Brazil | 0.258 | -0.034 | -0.009 | -0.157 | -0.907 | -0.002 | -0.005 | -0.055 |
| *Corythophora alta* | Brazil | 0.121 | 0.081 | 0.191 | NA | -0.968 | 0.14 | NA | 0.031 |
| *Corythophora rimosa* | Brazil | -0.254 | -0.103 | -0.277 | NA | 0.515 | -0.002 | NA | 0.014 |
| *Croton matourensis* | Brazil | -0.235 | -0.082 | -0.246 | -0.063 | 0.085 | -0.118 | -0.047 | -0.089 |
| *Eschweilera pseudodecolorans* | Brazil | -0.177 | 0.061 | -0.099 | 0.009 | 0.321 | 0.23 | -0.004 | 0.01 |
| *Eschweilera wachenheimii* | Brazil | -0.166 | -0.067 | -0.3 | -0.019 | 0.595 | -0.168 | 0.029 | 0.012 |
| *Helianthostylis sprucei* | Brazil | 0.049 | 0.03 | 0.071 | NA | -0.142 | -0.013 | NA | 0.021 |
| *Helicostylis tomentosa* | Brazil | 0.25 | 0.344 | 0.242 | 0.169 | 0.113 | 0.155 | 0.07 | -0.069 |
| *Lecythis prancei* | Brazil | -0.241 | -0.126 | -0.129 | NA | 0.543 | -0.165 | NA | 0.008 |
| *Micropholis guyanensis* | Brazil | -0.142 | -0.039 | -0.256 | 0.053 | -0.099 | -0.121 | -0.054 | 0.012 |
| *Protium hebetatum* | Brazil | 0.155 | 0.067 | 0.069 | -0.052 | -0.949 | 0.021 | -0.001 | 0.004 |
| *Rinorea racemosa* | Brazil | -0.04 | -0.07 | -0.057 | 0.109 | 0.068 | -0.039 | -0.024 | -0.012 |
| *Scleronema micranthum* | Brazil | 0.253 | 0.132 | 0.327 | 0.03 | 0.217 | 0.064 | 0.024 | 0.021 |
| *Swartzia recurva* | Brazil | -0.092 | 0.007 | 0.14 | NA | 0.482 | 0.179 | NA | 0.031 |
| *Tetragastris panamensis* | Brazil | 0.304 | 0.149 | 0.05 | -0.066 | 0.313 | 0.139 | 0.01 | 0.029 |
| *Zygia racemosa* | Brazil | 0.112 | 0.048 | -0.151 | NA | 0.099 | 0.053 | NA | 0.057 |
| *Bocoa prouacensis* | French Guiana | -0.093 | -0.157 | -0.082 | 0.216 | 0.032 | 0.049 | -0.057 | 0.098 |
| *Carapa surinamensis* | French Guiana | 0.545 | 0.102 | 0.108 | -0.104 | 0.264 | 0.166 | 0.04 | -0.078 |
| *Dicorynia guianensis* | French Guiana | -0.407 | -0.052 | -0.482 | -0.103 | -0.088 | -0.009 | 0.04 | -0.006 |
| *Iryanthera sagotiana* | French Guiana | 0.027 | -0.016 | 0.214 | -0.07 | 0.495 | -0.069 | -0.007 | -0.027 |
| *Lecythis persistens* | French Guiana | -0.194 | -0.289 | -0.264 | -0.143 | 0.442 | -0.223 | -0.033 | 0.065 |
| *Licania heteromorpha* | French Guiana | -0.018 | -0.202 | 0.087 | -0.028 | 0.259 | -0.111 | -0.039 | 0.011 |
| *Oxandra asbeckii* | French Guiana | 0.294 | 0.224 | 0.116 | 0.154 | -0.727 | -0.026 | -0.039 | 0.083 |
| *Celtis shippii* | Peru | 0.064 | -0.158 | 0.238 | NA | -0.344 | -0.031 | -0.031 | -0.009 |
| *Clarisia racemosa* | Peru | -0.199 | 0.128 | 0.109 | 0.057 | 0.238 | 0.177 | 0.01 | -0.018 |
| *Guarea macrophylla* | Peru | -0.021 | -0.125 | -0.119 | NA | -0.118 | -0.114 | 0.001 | 0.044 |
| *Iryanthera juruensis* | Peru | -0.204 | -0.242 | 0.029 | 0.01 | 0.134 | 0.002 | -0.005 | -0.031 |
| *Iryanthera laevis* | Peru | -0.022 | -0.068 | 0.067 | -0.003 | 0.022 | 0.155 | 0.004 | -0.059 |
| *Leonia glycycarpa* | Peru | -0.035 | -0.022 | 0.104 | NA | -0.048 | 0.026 | 0.043 | -0.03 |
| *Matisia bicolor* | Peru | 0.337 | 0.205 | 0.252 | NA | -0.38 | 0.104 | 0.044 | -0.042 |
| *Neea divaricata* | Peru | 0.108 | 0.348 | 0.248 | 0.108 | -0.172 | 0.01 | 0.043 | -0.003 |
| *Oxandra acuminata* | Peru | 0.303 | 0.144 | 0.153 | 0.022 | -0.173 | 0.172 | -0.063 | 0.011 |
| *Pourouma minor* | Peru | -0.032 | 0.022 | 0.065 | NA | 0.08 | -0.118 | 0.002 | 0.006 |
| *Pouteria franciscana* | Peru | -0.062 | 0.099 | -0.028 | NA | -0.04 | -0.052 | 0.013 | 0.038 |
| *Pouteria torta* | Peru | -0.446 | -0.142 | -0.357 | NA | -0.116 | -0.102 | -0.014 | 0.043 |
| *Pseudolmedia laevigata* | Peru | 0.227 | 0.035 | -0.073 | 0.136 | 0.007 | 0.062 | 0.026 | -0.038 |
| *Quararibea wittii* | Peru | 0.166 | 0.232 | 0.36 | NA | -0.596 | -0.05 | 0.01 | -0.038 |
| *Roucheria punctata* | Peru | 0.058 | -0.128 | 0.081 | -0.069 | 0.056 | -0.192 | -0.033 | 0.048 |
| *Tachigali polyphylla* | Peru | -0.118 | -0.109 | -0.136 | 0.075 | -0.096 | -0.01 | -0.04 | 0.063 |
| *Tetragastris altissima* | Peru | 0.365 | 0 | -0.029 | -0.092 | 0.229 | -0.012 | -0.009 | -0.037 |
| *Unonopsis matthewsii* | Peru | -0.375 | -0.177 | 0.051 | NA | -0.142 | -0.138 | 0.004 | -0.092 |
| *Virola sebifera* | Peru | -0.282 | -0.126 | -0.082 | -0.168 | 0.056 | -0.054 | -0.037 | -0.089 |
| *Brosimum rubescens* | Brazil and Peru | 0.103 | 0.114 | 0.017 | -0.022 | 0.041 | -0.072 | 0.025 | -0.021 |
| *Eschweilera coriacea* | Brazil and Peru | -0.21 | -0.107 | -0.332 | -0.075 | 0.52 | -0.004 | 0.015 | 0.02 |
| *Minquartia guianensis* | Brazil and Peru | -0.073 | -0.149 | -0.153 | 0.058 | -0.047 | 0.02 | -0.003 | 0.055 |
| *Pseudolmedia laevis* | Brazil and Peru | 0.098 | 0.064 | -0.063 | NA | -0.532 | 0.04 | 0.021 | -0.024 |
| *Siparuna decipiens* | Brazil and Peru | 0.01 | 0.167 | 0.45 | 0.13 | 0.348 | 0.085 | 0.031 | -0.006 |
| *Symphonia globulifera* | Brazil, Peru and French Guiana | -0.068 | -0.012 | -0.115 | -0.099 | 0.106 | -0.035 | 0.041 | 0.035 |

Elements on a volume basis, water content per unit tissue volume (*Φ*) and wood density (*ρ*) were log10 transformed prior to analyses.

**Table S3.** Arithmetic mean values and range between brackets of each assessed trait per plot. Significant differences as obtained with the Tukey’s ‘Honest Significant Difference’ method (*P* < 0.05) are indicated by different superscripts. Plots with non-significant differences show same superscript. *ρ* = branch wood density; *Φ* = branch volumetric water content. Calcium, magnesium, potassium, sodium, nitrogen and phosphorus concentrations are shown expressed on both a branch mass basis (subscript “m”) and branch volume basis (subscript “=v”). N.D = not determined.

|  | **Brazil** | | **French Guiana** | | | **Peru** | | |
| --- | --- | --- | --- | --- | --- | --- | --- | --- |
| **Trait** | **DUK-26** | **MAN-12** | | **GFX-19** | **GFX-07** | | **TAM-07** | **CUZ-03** |
| *ρ* (kg m^-3^) | 696^a^  (516 - 809) | 552^b^  (–480-908) | | 607^b,c^  (413 - 757) | 657^a,b^  (563 - 876) | | 552^,d^  (316 - 768) | 532^d^  (393 - 642) |
| *Φ* (m^3^ m^-3^) | N.D. | 0.448^c^  (0.316-0.589) | | 0.487^b,c^  (0.384-0.628) | 0.455^c^  (0.328-0.551) | | 0.536^a^  (0.337-0.762) | 0.517^a,b^  (0.414 0.647) |
| [Ca]_m_ (mmol g^-1^) | 0.02^d^  (–0.01-0.07) | 0.03^c,d^  (–0.01-0.08) | | 0.07^a,b^  (0.01-0.24) | 0.08^a,b,c^  (0.01-0.42) | | 0.02^c^  (–0.01-0.09) | 0.12 ^a^  (–0.01-0.51) |
| [Ca]_v_ (mol m^-3^) | 13.0^b^  (3.7 - 46.5) | 17.7^b^  (4.7 - 50.3) | | 39.6^a,b^  (3.1 - 106.7) | 49.5^a^  (5.3 -237.1) | | 13.7^a,b^  (3.3 - 55.0) | 57.9^a^  (8.6 -188.9) |
| [Mg]_m_ (mmol g^-1^) | 0.02^b^  (0-0.06) | 0.02^b^  (0-0.07) | | 0.02^b^  (0-0.06) | 0.02^b^  (0-0.04) | | 0.03^b^  (0-0.09) | 0.05^a^  (0.01-0.15) |
| [Mg]_v_ (mol m^-3^) | 13.8^b^  (3.3 - 38.9) | 14.6^b^  (2.6 - 49.8) | | 13.5^b^  (1.6 - 31.8) | 10.1^b^  (1.8 - 22.0) | | 14.6^ab^  (2.8- 49.1) | 22.5^a^  (2.6 - 63.8) |
| [K]_m_ (mmol g^-1^) | 0.038^c^  (0.01-0.15) | 0.04^c^  (0.01-0.1) | | 0.05^bc^  (0.01-0.13) | 0.04^c^  (0.02-0.08) | | 0.07^b^  (–0.02-0.2) | 0.09^a^  (0.03-0.22) |
| [K]_v_ (mol m^-3^) | 22.0^b^  (8.8 -78.5) | 24.6^b^  (9.9 - 58.0) | | 28.6^a,b^  (9.6 - 65.4) | 28.8^a,b^  (13.5 - 56.0) | | 36.5^a^  (11.2 -114) | 47.6^a^  (16.1 – 105) |
| [Na]_m_ (mmol g^-1^) | 0.02^a^  (0-0.08) | 0.02^a^  (0-0.09) | | 0.03^a^  (0-0.07) | 0.03^a^  (0.01-0.06) | | 0^b^  (0-0) | 0^b^  (0-0) |
| [Na]_v_ (mol m^-3^) | 16.5^a^  (0.59 -48.9) | 14.8^a^  (0.41 - 53.5) | | 16.3^a^  (0.73 - 37.0) | 19.7^a^  (7.9- 44.2) | | 1.02^b^  (0.35 - 2.02) | 0.47^c^  (0.08 - 1.7) |
| [N]_m_ (mmol g^-1^) | N.D. | 0.41 ^a^  (0.18-0.84) | | 0.27^b,c^  (0.13-0.55) | 0.18^c^  (0.1-0.32) | | 0.41^a^  (0.21-0.85) | 0.28^b^  (0.12-0.52) |
| [N]_v_ (mol m^-3^) | N.D. | 268^a^  (113 - 461) | | 164^b^  (68 - 351) | 123^b^  (67 – 284) | | 222^a^  (98- 412) | 132^b^  (52.1- 221) |
| [P]_m_ (mmol g^-1^) | 0.01^c^  (0-0.03) | 0.01^c^  (0-0.01) | | 0.01^c^  (0-0.03) | 0^c^  (0-0.01) | | 0.01^b^  (0-0.07) | 0.04^a^  (0.01-0.09) |
| [P]_v_ (mol m^-3^) | 4.6^c^  (1.6 -18.0) | 3.4^c^  (1.5 - 7.0) | | 3.4^c^  (1.2 - 11.6) | 3.0^c^  (1.3 - 5.5) | | 7.9^b^  (2.06 - 35.7) | 21.7^a^  (4.6 - 43.6) |

**Table S4** Mixed effect modelling of the relationships between nutrients on a dry mass basis *Θ*_m_ and wood density ***ρ*.**

| Nutrient | [Ca]_m_ | [K]_m_ | [Mg]_m_ | [N]_m_ | [Na]_m_ | [P]_m_ |
| --- | --- | --- | --- | --- | --- | --- |
| **Fixed effects** | | | | | | |
| Intercept (γ_00_) | 0.45 | 2.96 | 1.71 | 1.00 | 1.80 | 1.82 |
| Slope (γ_10_) | -0.69 | **-1.55** | **-1.22** | -0.55 | **-1.45** | **-1.41** |
| *P* | 0.092 | <0.001 | 0.001 | 0.054 | 0.004 | <0.001 |
| $R_{M}^{2}$ | 0.01 | 0.17 | 0.08 | 0.04 | 0.02 | 0.06 |
| $R_{C}^{2}$ | 0.74 | 0.70 | 0.59 | 0.68 | 0.91 | 0.77 |
| **Random effects** | | | | | | |
| Species ($\sigma_{s}^{2}$) | 0.062 | 0.026 | 0.049 | 0.014 | 0.166 | 0.021 |
| Plot ($\sigma_{p}^{2}$) | 0.070 | 0.012 | 0.003 | 0.017 | 0.382 | 0.093 |
| Residual ($\sigma_{r}^{2}$) | 0.047 | 0.022 | 0.041 | 0.016 | 0.057 | 0.037 |

All parameters were estimated using restricted maximum likelihood (REML). $R_{M}^{2}$=marginal *R*^2^; $R_{C}^{2}$=conditional *R*^2^. Significant relationships (*P* < 0.05) are in bold. Random effects present the variance (σ^2^) of the random intercepts (Eqn. 6).

**Table S5** Estimates of relationships between nutrients on a volume basis with wood density *ρ* and water content *Φ* using ordinary least squares (OLS).

| *Θ*_v_ (mol m^-3^) *vs* Wood density *ρ* (kg m^-3^) | | | | | | |
| --- | --- | --- | --- | --- | --- | --- |
| Nutrient | [Ca]_v_ | [K]_v_ | [Mg]_v_ | [N]_v_ | [Na]_v_ | [P]_v_ |
| Intercept | 5.61 | 5.54 | 3.95 | -0.51 | -14.66 | 8.07 |
| Slope | **-1.57** | **-1.47** | **-1.02** | **1.00** | **5.44** | **-2.63** |
| *P* | <0.001 | <0.001 | <0.001 | <0.001 | <0.001 | <0.001 |
| $R^{2}$ | 0.08 | 0.18 | 0.06 | 0.13 | 0.26 | 0.24 |
| *Θ*_v_ (mol m^-3^) *vs* Water content *Φ* (m^3^ m^-3^) | | | | | | |
| Nutrient | [Ca]_v_ | [K]_v_ | [Mg]_v_ | [N]_v_ | [Na]_v_ | [P]_v_ |
| Intercept | 1.56 | 2.08 | 1.49 | 2.26 | -0.79 | 1.57 |
| Slope | 0.80 | **1.93** | **1.21** | -0.04 | **-3.53** | **2.48** |
| *P* | 0.109 | <0.001 | 0.002 | 0.896 | <0.001 | <0.001 |
| $R^{2}$ | 0.02 | 0.25 | 0.06 | 0.00 | 0.09 | 0.15 |

**Table S6.** Mixed effect modelling of the relationships between nutrients on a dry mass basis *Θ*_m_ and tissue water content *Φ*.

| Nutrient | [Ca]_m_ | [K]_m_ | [Mg]_m_ | [N]_m_ | [Na]_m_ | [P]_m_ |
| --- | --- | --- | --- | --- | --- | --- |
| **Fixed effects** | | | | | | |
| Intercept (γ_00_) | -1.11 | -0.80 | -1.26 | -0.24 | -2.00 | -1.72 |
| Slope (γ_10_) | **0.94** | **1.57** | **1.25** | **0.91** | 0.88 | **1.08** |
| *P* | 0.030 | <0.001 | 0.001 | 0.001 | 0.082 | 0.003 |
| $R_{M}^{2}$ | 0.02 | 0.15 | 0.06 | 0.08 | 0.00 | 0.03 |
| $R_{C}^{2}$ | 0.73 | 0.71 | 0.65 | 0.70 | 0.91 | 0.78 |
| **Random effects** | | | | | | |
| Species (σ_s_^2^) | 0.065 | 0.025 | 0.059 | 0.015 | 0.132 | 0.019 |
| Plot (σ_p_^2^) | 0.066 | 0.013 | 0.007 | 0.014 | 0.477 | 0.123 |
| Residual (σ_r_^2^) | 0.051 | 0.019 | 0.039 | 0.014 | 0.061 | 0.040 |

All parameters were estimated using restricted maximum likelihood (REML). $R_{M}^{2}$=marginal *R*^2^; $R_{C}^{2}$=conditional *R*^2^. Significant relationships (*P* < 0.05) are in bold. Random effects present the variance (σ^2^) of the random intercepts (Eqn. 6).

**References**

Epron D., Bosc A., Bonal D. & Freycon V. (2006) Spatial variation of soil respiration across a topographic gradient in a tropical rain forest in French Guiana. *Journal ofTropical Ecology* **22**, 565–574.

Hättenschwiler S. & Jørgensen H.B. (2010) Carbon quality rather than stoichiometry controls litter decomposition in a tropical rain forest. *Journal of Ecology* **98**, 754–763.

Hijmans R.J., Cameron S.E., Parra J.L., Jones P.G. & Jarvis A. (2005) Very high resolution interpolated climate surfaces for global land areas. *International Journal of Climatology* **25**, 1965–1978.

Quesada C.A., Lloyd J., Schwarz M., Patiño S., Baker T.R., Czimczik C.I., … Paiva R. (2010) Variations in chemical and physical properties of Amazon forest soils in relation to their genesis. *Biogeosciences* **7**, 1515–1541.
