# Supplementary material for: Wood Nutrient-Water-Density Linkages Are Influenced by Both Species and Environment
Source: Front Plant Sci. 2022 Apr 4;13:778403. doi: 10.3389/fpls.2022.778403 (PMC9014131; doi:10.3389/fpls.2022.778403)
Supplement: Supplementary file 2 [file Data_Sheet_2.docx]

**Supporting Information II for:**

Wood nutrient-water-density linkages are influenced by both species and environment

**SI1. Relationships in a mass basis *vs* volume basis**

The choice of which units to use when quantifying a plant trait is not necessarily a trivial matter, and with this already having been a matter of some discussion in ecological circles, especially with some challenges having been made as to the validity of widely touted leaf trait associations, and with implications that have extended to their associated ecological significance. For example, two independent groups have challenged the rationale of the mass-based leaf economic spectrum concept arguing that correlations between area based photosynthetic capacity and leaf structure are in fact spurious (Lloyd, Bloomfield, Domingues & Farquhar 2013; Osnas, Lichstein, Reich & Pacala 2013). These observations triggered the discussions on how to interpret such correlations with no current consensus (Westoby, Reich & Wright 2013; Poorter, Lambers & Evans 2014). Indeed, depending on the units being used, the inferred nature and significance of the associations can sometimes be remarkably different.

Thus, it is not a trivial question to ask whether correlations between *ρ*, *Φ* and nutrients should be considered on a mass *vs* volume basis? One way to look at the problem is to argue that correlations between *ρ* and *Θ*_m_ implicitly suggest that any changes in wood density should affect nutrient amounts when expressed relative to the woody mass present. Thus, a negative correlation between *ρ* and many *Θ*_m_ suggests that, for a given volume of wood, an increased amount of woody mass will also have a lower amount of nutrient within. Conversely, a positive relationship between *Φ* and *Θ*_m_ can be interpreted as, for a given volume of wood, a larger volume of water being associated with a greater amount of nutrients within the woody mass itself. Such a relationship does not, therefore, imply a direct association between the nutrients and the volume of water in the woody tissues. But rather, it reflects on how the mass of nutrients should change in relation to the mass of wood present when water content varies. In contrast, when both nutrient and water are expressed on a volume basis, the associations are more directly and easily interpretable. This is because as all units are of comparable dimensions in accordance with the general concept of “similarity scaling” as widely applied in the physical sciences where the general desirability of expressing all variables in the same dimensions are well recognised (Barenblatt 1996). Given that wood density is by its very nature a plant trait expressed on volume basis then the logical practice should be to examine wood and water contents and nutrient concentrations on the same basis when analysing inter-relationships between these traits. The use of these traits in the same volumetric context can further be conceptualised as describing in a simple way the relative distributions of dry matter, water and nutrients distribution within the same finite tissue volume.

**SI2. Relationships beteween the regression slope for the log-transformed mass and volume -based cases.**

Here, it was found that the although with the same standard error, the slope estimates of the (log-transformed) volume-based elemental concentrations when expressed as a function of the (log-transformed) wood density were always greater than the mass-based relationships by the amount of (exactly) 1.00. That is to say for the relationship = *a + b*_v_where the “ℓ” superscripts of the remind us that the variables have been transformed then it must be the case that

In order to establish why this should be so, we first note that as pointed out by Chayes (1971)

variances can be estimated parameters defining the nature of the regression relationships. For example, in the simple linear regression equation *y = a + bx*, then the least squares estimate of the slope is simply the ratio of the (*x,*y) covariance, *C*(*x,y*), to the variance of *x*; *viz.*

$$b=\frac{C(x,y)}{\sigma_{x}^{2}}\begin{matrix} & & \end{matrix}.\begin{matrix} & & \end{matrix}(A1)$$

We start with the simple observation that as ${}\Theta_{\text{v}}=\Theta_{\text{m}}\rho$ then$\log(\Theta_{\text{v}})=\log(\Theta_{\text{m}}\rho)$ and thus . This means that an examination of as a function of is also an examination of () as a function of  and thus we have a situation directly analgous to the area versus mass based leaf trait correlation inter-relatioships as analysed by (Lloyd *et al.* 2013).

Therefore, using the same theoretial approach as in the Appendix of that classic tome, we start with the simpler correlation, for which in formal statistical terms we can define the covariance as

where *E* denotes the expected value and with = *E*() and= *E*(). Likewise, the associated standard deviations can be formally defined through the poulation *variances* (*V*) as

Noting the overlap in the expectation terms between Eqn A3 and Eqn A4, for ease of presentation later we define two *deviations*

From which it then follows that

Using standard, small sample statistical theoretical approaches – and also pointing out that what follows may be found expressed in general terms on page 25 of Chayes (1971) – for expressed in terms of its mean (*μ*) and deviations from that mean (*δ*) then:

and thus the expectation is

Likewise, with

It then follows that

For ^ℓ^*Θ*_v_ the situation is, however, less straight-forward, as

so that

Then

and the product of the deviances is then

Converting to expectations and by reference to Eqn A8 and Eqn A10 it then follows that

and by refetrence to Eqn A2 this then means that

Or expressed another way

$$b_{\text{m}}\equiv b_{\text{v}}-1\begin{matrix} & \end{matrix},\begin{matrix} & & \end{matrix}(\text{A17})$$

This simple mathematical proof provides an explanation as to, irrespective of whether or not increases or declines with, that the slope of the relationship is always greater by a factor of exactly 1.0 as compared to the equivalent *vs.* relationship.

To explain the identical standard errors, we start of with a general equation for the unbiased estimator of the error in the slope of *b* as can be obtained in any basic text regression book

$$\sigma^{2}(b)=\frac{MSE}{\sigma^{2}(x)}\begin{matrix} & & \end{matrix},\begin{matrix} & & \end{matrix}(\text{A18})$$

where *MSE* is the error mean square, estimable as

$$MSE=\frac{SSE}{n-2}\begin{matrix} & & \end{matrix},\begin{matrix} & & \end{matrix}\text{(A19})$$

with *SEE* being the error sum of squares which can be written in many ways, the most informative of which for our purposes is

$$SEE=\mathop{}\limits\sigma_{\text{y}}^{2}\mathop{}\limits-\mathop{}\limits\frac{\left[C(x,y)\right]^{2}}{\sigma_{x}^{2}}\begin{matrix} & & \end{matrix},\begin{matrix} & & \end{matrix}(\text{A20})$$

and thus combining Eqn A17 through A20 one can estimate the standard error of *b* as

$$\begin{matrix} s(b)=\mathop{}\limits\sqrt{\frac{\sigma_{\text{y}}^{2}\mathop{}\limits-\mathop{}\limits\frac{\left[C(x,y)\right]^{2}}{\sigma_{x}^{2}}}{\sigma_{x}^{2}(n-2)}}\begin{matrix} & & \end{matrix}.\begin{matrix} & & \end{matrix}(\text{A21}) \\ \end{matrix}$$

Taking again first our area-based trait, *Θ*_m_ the a substitution of the relevant terms leads to

And the equivalent equation for *b*_v_ is

Our task is to see if we can derive A22a from A22b which would mean that the regression slope standard errors of the mass *vs.* volume based trait expessions must be identical.

We first find need an identity for expressed in terms of which turns out to be simply enough as from Eqn 13

whch means that

and so by substitution into Eqn A22b

and then with reference to Eqn A15 to replace the term with one involving just and its associated variances

from which of follows that

which simplifies to

and which then further reduces to the exact same form of Eqn A22a *viz.*

This shows that despite the different variance and covariance terms within Eqn 22a *vs.* Eqn 22b they are mathematically equivalent and so $s(b_{\text{v}})\equiv s(b_{\text{m}})$. This contrasts with the situation for the slopes themsleves as shown through the derivation of Eqn A17 are inevitably different. Thus, with the *t* test of regression slope signifance being most simply estimated as

$$t_{b_{\text{x}}}=\frac{b_{\text{x}}}{s(b_{\text{x}})}\begin{matrix} & & \end{matrix},\begin{matrix} & & & \end{matrix}(\text{A29})$$

then this inevitably means that the *b*_v_ slope should be negative then the *b*_m_ slope will be, not only exactly one unit more negative, but also inevitably of a greater statistical significance. For 0.0 < *b*_v_ < 1.0 then *b*_v_ and *b*_m_ will inevitably be of different signs (with *b*_m_ necessarily negative) and for *b*_v_ > 1.0 (and thus *b*_m_ also positive) then the apparent significance of the regression slope of any entity that increases as a function of wood density on both a mass a volumetric basis will actually be greater for *b*_v_ than *b*_m_. This was not the case for any of the entities studied here, but could be the case for wood lignin content for example (Rana, Langenfeld-Heyser, Finkeldey & Polle 2010).

**References**

Barenblatt G.I. (1996) *Scaling, self-similarity, and intermediate asymptotics: dimensional analysis and intermediate asymptotics*. Cambridge University Press.

Chayes F. (1971) *Ratio correlation: a manual for students of petrology and geochemistry*. University of Chicago Press.

Lloyd J., Bloomfield K., Domingues T.F. & Farquhar G.D. (2013) Photosynthetically relevant foliar traits correlating better on a mass vs an area basis: Of ecophysiological relevance or just a case of mathematical imperatives and statistical quicksand? *New Phytologist* **199**, 311–321.

Osnas J.L.D.D., Lichstein J.W., Reich P.B. & Pacala S.W. (2013) Global leaf trait relationships: Mass, area, and the leaf economics spectrum. *Science* **340**, 741–744.

Poorter H., Lambers H. & Evans J.R. (2014) Trait correlation networks: A whole-plant perspective on the recently criticized leaf economic spectrum. *New Phytologist* **201**, 378–382.

Rana R., Langenfeld-Heyser R., Finkeldey R. & Polle A. (2010) FTIR spectroscopy, chemical and histochemical characterisation of wood and lignin of five tropical timber wood species of the family of Dipterocarpaceae. *Wood Science and Technology* **44**, 225–242.

Westoby M., Reich P.B. & Wright I.J. (2013) Understanding ecological variation across species: area- based vs mass-based expression of leaf traits. *New Phytologist* **199**, 322–323.
