# Supplementary material for: Wood Nutrient-Water-Density Linkages Are Influenced by Both Species and Environment
Source: Front Plant Sci. 2022 Apr 4;13:778403. doi: 10.3389/fpls.2022.778403 (PMC9014131; doi:10.3389/fpls.2022.778403)
Supplement: Supplementary file 3 [file Data_Sheet_3.docx]

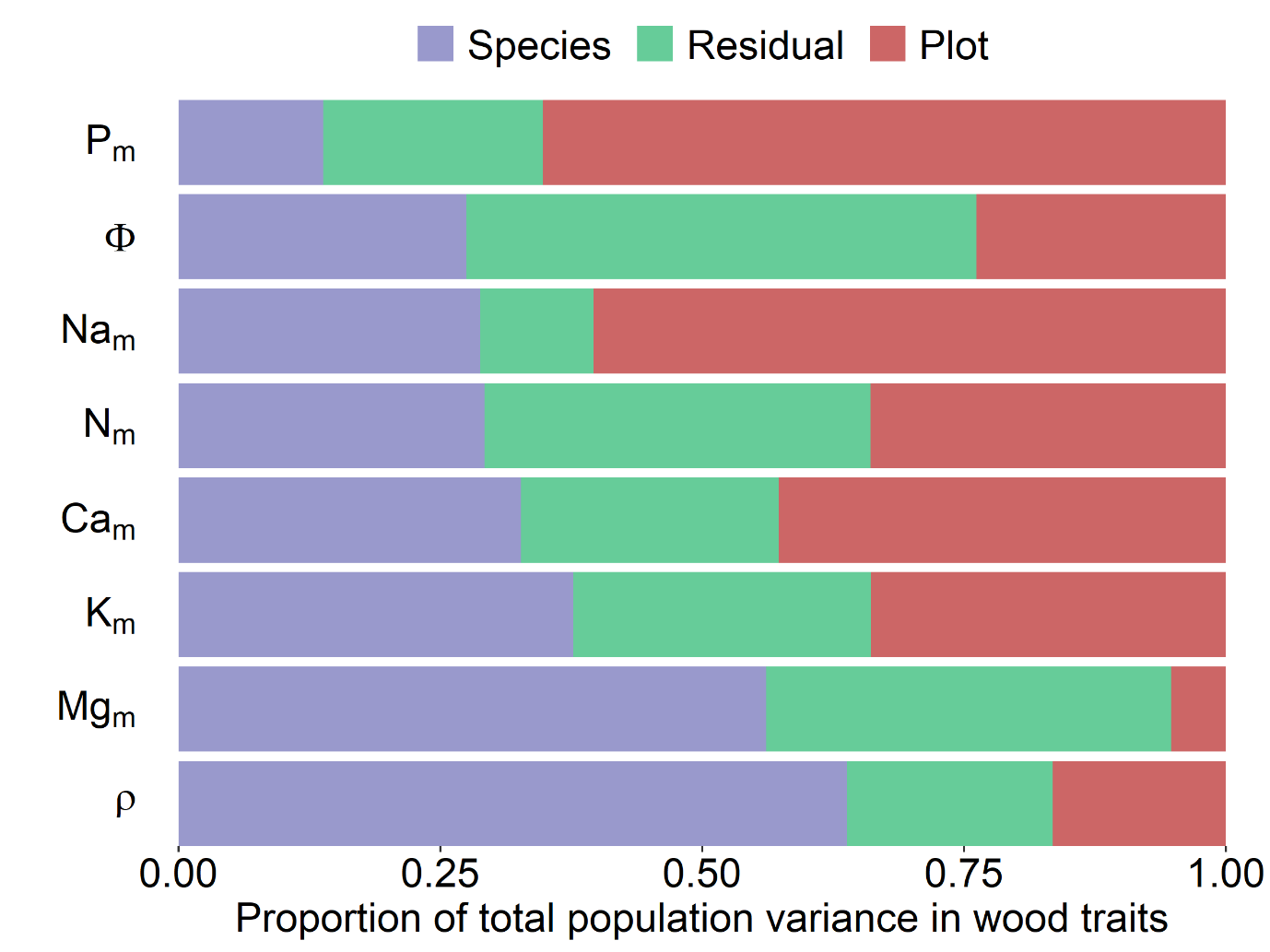


**Figure S1.** Partitioning of the total variance for each woody property (y axis) into species, environmental (plot) and error (residual) components. Traits have been ranked according to the relative contribution of the species-associated variance component. All values were log_10_ transformed prior to analysis and with nutrients on a mass basis being used. Subscript “m” indicates that concentrations are on a mass basis. *ρ* = Wood density.

.


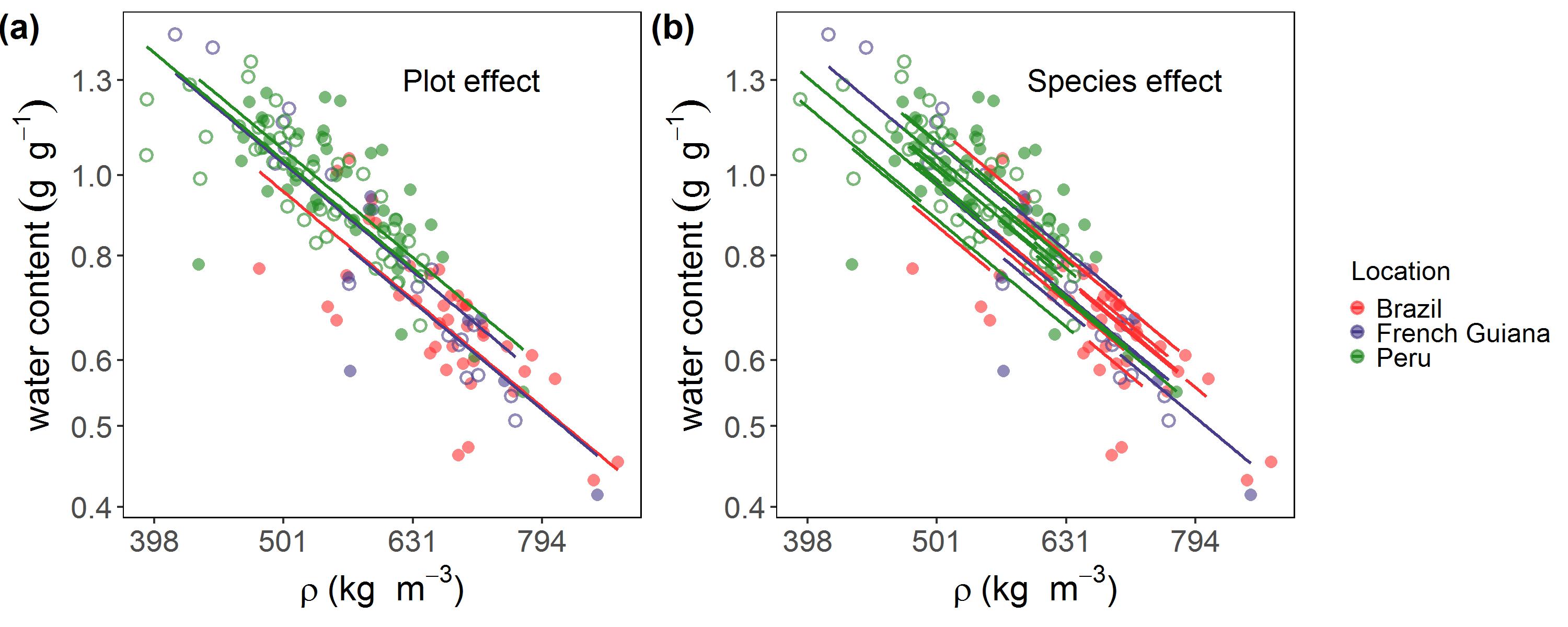


**Figure S2** Relationships between wood density (*ρ*) and water content on a mass basis. Lines are predicted estimates of mixed models for the species and plot effects (Eqn. 6). Open and closed circles represent the plots within each location.


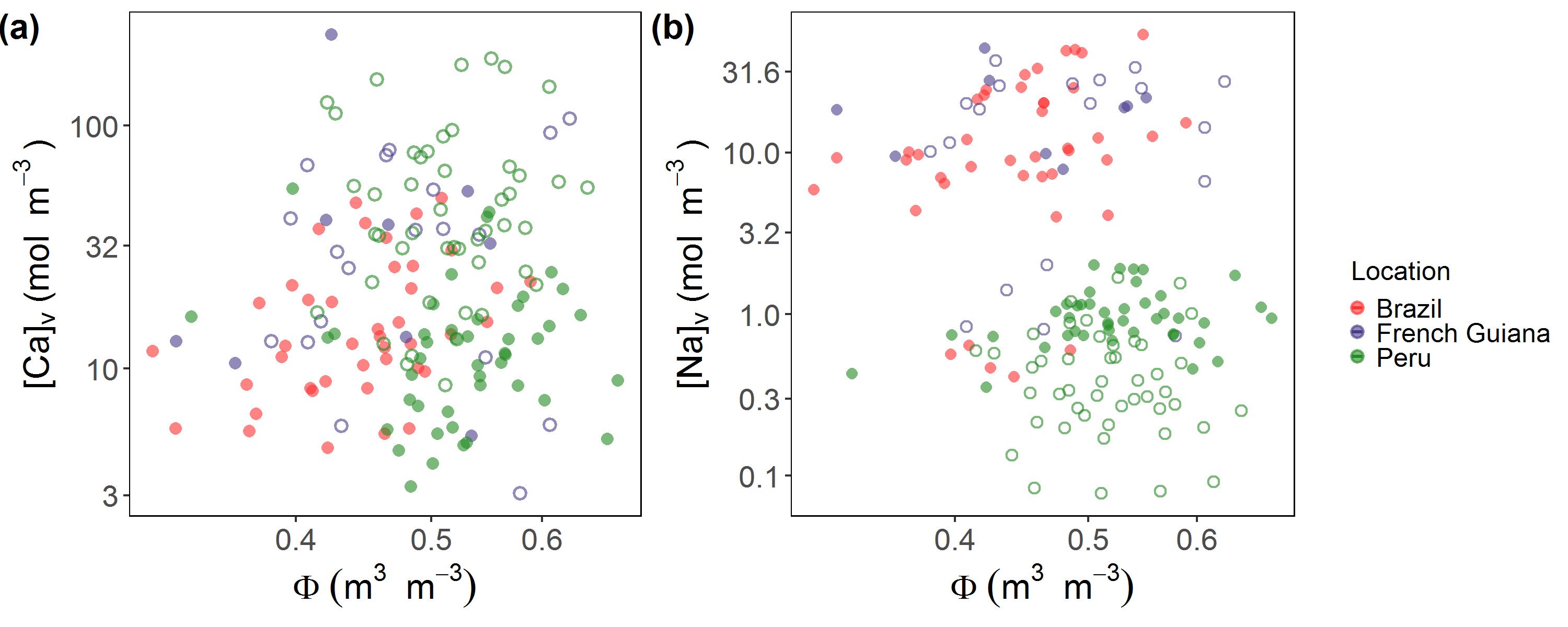


**Figure S3.** Relationships between water content (*Φ*) and **a)** calcium and **b)** sodium on a volume basis (*Θ*_v_). Open and closed circles represent the plots within each location


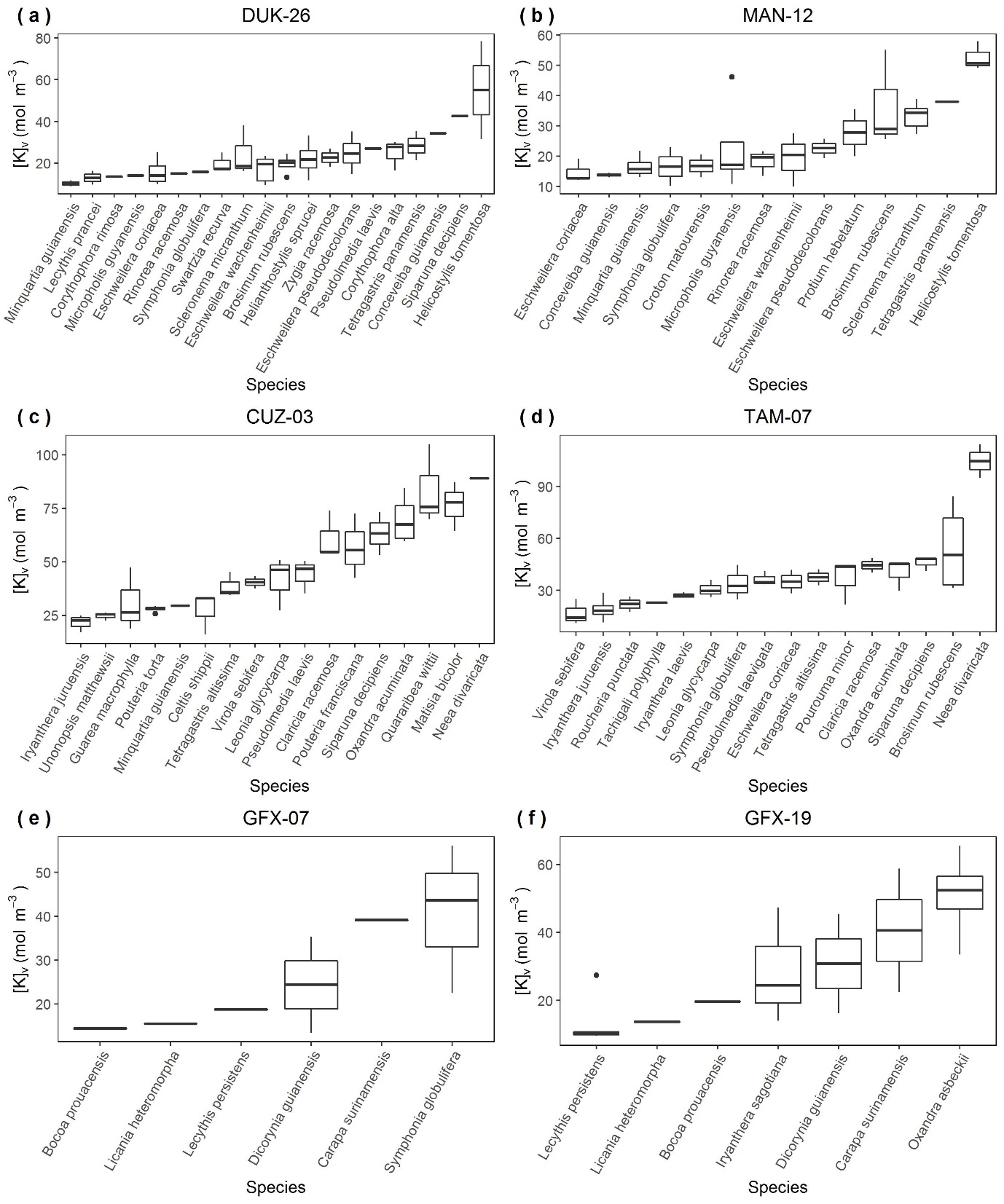


**Figure S4.** Variation of K wood tissue concentration on a volumetric basis for site in Brazil **(a)** and **(b),** Peru **(c)** and **(d)** and French Guiana **(e)** and **(f)**


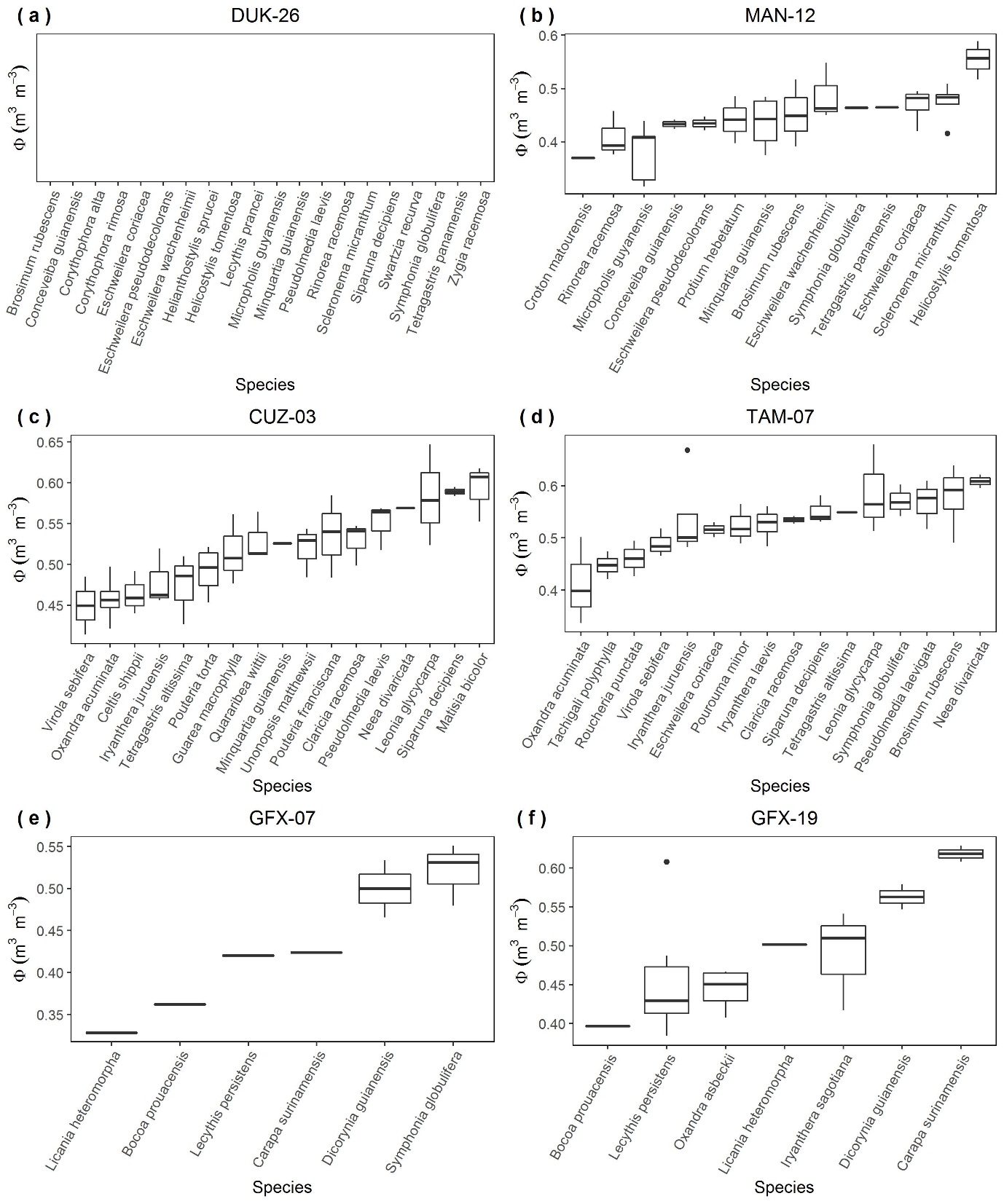


**Figure S5.** Variation in water content in wood on a volumetric basis for the sites in Brazil **(a)** no data available for this site**; (b),** Peru **(c)** and **(d)** and French Guiana **(e)** and **(f).**
